# Structural Insights on Tiny Peptide Nucleic Acid (PNA) Analogues of miRNA-34a: An *in silico* and Experimental Integrated Approach

**DOI:** 10.3389/fchem.2020.568575

**Published:** 2020-11-23

**Authors:** Maria Moccia, Flavia Anna Mercurio, Emma Langella, Valerio Piacenti, Marilisa Leone, Mauro F. A. Adamo, Michele Saviano

**Affiliations:** ^1^Institute of Crystallography, National Research Council, Department of Chemical Sciences and Materials Technologies, Bari, Italy; ^2^Institute of Biostructures and Bioimaging, National Research Council, Naples, Italy; ^3^Royal College of Surgeons in Ireland, Department of Pharmaceutical and Medicinal Chemistry, Dublin, Ireland

**Keywords:** PNA, miRNA-34a, NMR, tumor suppressor, molecular dynamics

## Abstract

In the present work, structural features of the interaction between peptide nucleic acid (PNA)-based analogs of the tumor-suppressor microRNA-34a with both its binding sites on MYCN mRNA were investigated. In particular, the region from base 1 to 8 (“seed” region) of miR-34a was reproduced in the form of an 8-mer PNA fragment (tiny PNA), and binding to target 3'UTR MYCN mRNA, was studied by a seldom reported and detailed NMR characterization, providing evidence for the formation of anti-parallel duplexes with a well-organized structural core. The formation of PNA-3'UTR duplexes was also confirmed by Circular Dichroism, and their melting curves were measured by UV spectroscopy. Nevertheless, this study offered a valuable comparison between molecular dynamics predictions and experimental evidence, which showed great correlation. Preliminary uptake assays were carried out in Neuroblastoma Kelly cells, using short peptide conjugates as carriers and FITC fluorescent tag for subcellular localization. Moderate internalization was observed without the use of transfecting agents. The reported results corroborate the interest toward the design and development of chimeric PNA/RNA sequences as effective RNA-targeting agents.

## Introduction

**Figure 1:**
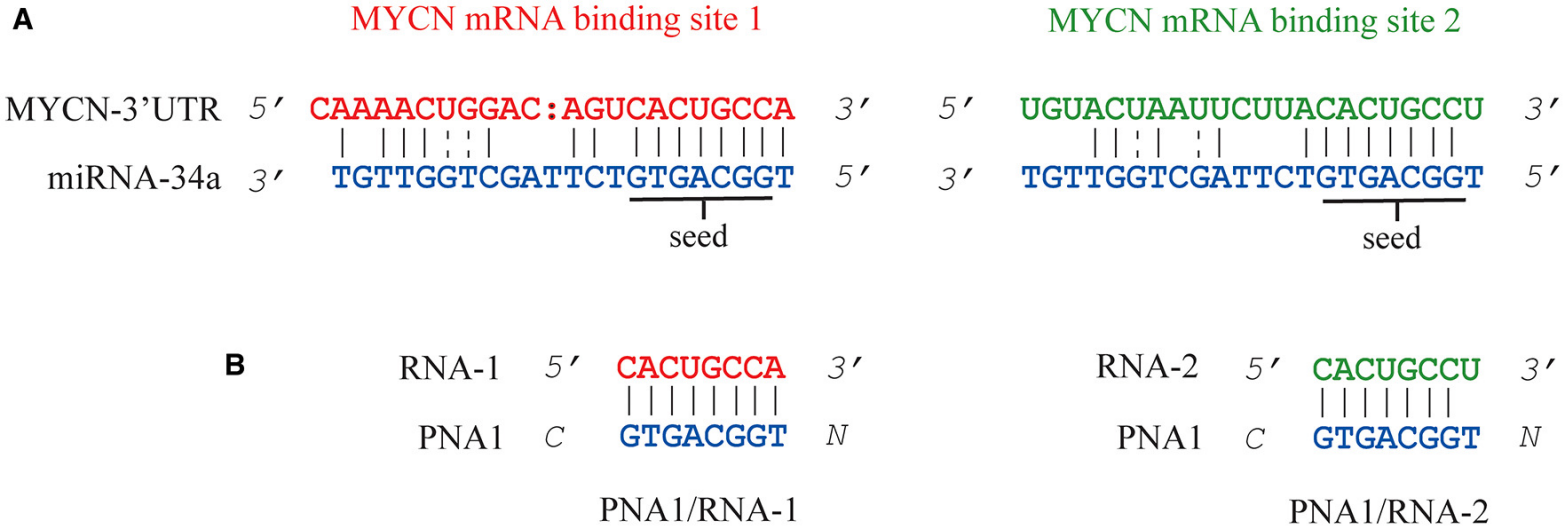
Representation of miRNA-34a binding sites on 3'UTR MYCN mRNA **(A)**. MYCN mRNA binding site 1 and binding site 2 are shown in red and green, respectively. The seed region is underlined. Sequences of the two hetero duplexes PNA1/RNA-1 and PNA1/RNA-2 studied in this work **(B)**.

In the last twenty years, small (19–25 nucleotides long), highly conserved, non-coding RNAs known as microRNAs (miRNAs) have emerged as a new family of cellular regulators (Bartel, 2009). The majority of miRNAs are transcribed from DNA sequences to generate a primary miRNA (pri-miRNA), which is processed into a precursor miRNA (pre-miRNA) and then in a mature miRNA (O'Brien et al., 2018). The miRNA duplex containing the guide and passenger strands not fully complementarily bound, is loaded into an AGO2 protein to form the RNA-induced silencing complex (RISC) (Iwasaki et al., 2010), which separates the RNA duplex, discarding the passenger strand and forming the mature RISC complex (Kobayashi and Tomari, 2016) with the guide miRNA strand. The miRNAs guide the RNA-induced silencing complex (RISC) to target 3'UTR mRNAs through the seed-pairing rule resulting in translation inhibition and/or RNA degradation (Iwakawa and Tomari, 2015). MiRNAs, have a crucial role in the regulation of mRNA translation in both physiological and pathological conditions. Because of their ability to fine-tune mRNA expression, miRNAs are commonly regarded as master modulators of the human genome. There is now ample evidence that the expression of individual or entire families of miRNAs is altered in many diseases, including cancer (Garzon et al., 2010). The study of miRNA expression profiling provides relevant information about tumor phenotype, progression, recurrence, metastasis as well as drug resistance (Stahlhut and Slack, 2013). Simultaneous over-expression and/or under-expression of different miRNAs is often related to pathological conditions and a signature miRNA pattern expression is recognizable in most cases (Croce, 2009). As a result, miRNAs may represent a very attractive class of molecules in drug development for a number of convenient features: (a) a single miRNA can regulate simultaneously different pathways by binding to the 3′UTR of multiple target genes (b); (Betel et al., 2010; Li and Zhang, 2015) once a miRNA sequence is identified and selected for drug development, the design and synthesis are relatively straightforward thanks to the peculiar small size (~22 nucleotides in length); (c) miRNAs are often conserved among species (Christopher et al., 2016); (d) miRNA-based drug delivery can be achieved *in vivo* by using different delivery platforms already approved for human use (Chang and Yeh, 2012; Rupaimoole and Slack, 2017). Two main therapeutic approaches are currently in use involving miRNAs depending on whether a particular miRNA expression needs to be downregulated (miRNA inhibition therapy) or upregulated by re-introducing the miRNA and restore lost functions (miRNA replacement therapy) (Rupaimoole et al., 2016; Mollaei et al., 2019). Oncogenic and over-expressed miRNAs in tumors can be inhibited by direct targeting with different approaches such as antisense oligonucleotides (ASOs strategy), anti-miRNA oligonucleotides (AMOs), miRNA antagomirs based on 2'-O-methyl (2′-OMe), 2′-O-methoxyethyl (2′-MOE), 2′-fluoro/2′-methoxyethyl (2′-F/MOE), bearing a phosphorothioate backbone which confers improved stability compared to native RNA sequences (Baumann and Winkler, 2015). Other DNA/RNA analogs, such as LNA (locked-nucleic-acids) and PNAs (peptide nucleic acids), have also been employed for miRNAs targeting thanks to their superior properties in terms of binding stability/affinity, chemical and enzymatic resistance (Lima et al., 2018). Indirect strategies, rely on the employment of virus-based constructs (Shah et al., 2016), such as adeno (Miyazaki et al., 2012), or lentiviral (Maegdefessel et al., 2012), to selectively transfer RNA-based drugs into the cells by packing a short hairpin RNA (shRNA) expressing artificial plasmids. Additional targeting approaches that may also be used to regulate miRNAs are: (1) miRNA sponges, (2) miRNA-zipper, (3) artificial ribonuclease, (4) target protector, (5) CRISPR/Cas9 gene-editing system, (6) small-molecules (To et al., 2020). A phosphorothioate-modified LNA antagomiR, Miravirsen, by Roche/Santaris Pharma, targets miR-122 to repress hepatitis C viral (HCV) infections (Lindow, 2012) and entered phase 2 clinical trials in 2017. Another LNA-based antagomirs of miR-155, which regulates differentiation and proliferation of blood and lymphoid cells, was employed to treat specific types of lymphoma and leukemia (Seto et al., 2018) and it is actually undergoing clinical trials in phase 1 and 2. In miRNA replacement therapy, tumor-suppressor miRNAs are reintroduced into the system to restore those lost functions resulting from downregulation of such key-miRNAs (Hosseinahli et al., 2018). In particular, synthetic miRNA mimics can be reintroduced in the system to act like endogenous mature double-stranded miRNA and restore/enhance miRNA function (Chen et al., 2015). Both double-stranded miRNA (miRNA-34a, let-7) (Johnson et al., 2005) and chemically modified single stranded miRNA mimics (miR-124, miR-122, miR-34a, and miR-21) were reported in literature (Matsui et al., 2016). Some promising miRNA mimic drugs such as MRG-201, mesomiR 1, and miR-16 mimics are currently being studied in Phase 1 clinical trials (Bajan and Hutvagner, 2019). In order to expand this chemical space, we have recently proposed the use of peptide nucleic acids (PNAs) as miRNA-34a analogs (Piacenti et al., 2019). PNAs are DNA/RNA analogs possessing a pseudo-peptide backbone made of N-(2 aminoethyl) glycine *(aeg*) motif to replace the sugar phosphate one (Nielsen et al., 1991). These chemical entities may provide a convenient platform for the preparation of specific sequences, as they combine desirable hybridization properties (Egholm et al., 1993; Nielsen et al., 1993; Moccia et al., 2016), robust synthesis, ease of conjugation to cell penetrating peptides (CPP) (Lee et al., 2014), and high biochemical stability (Demidov et al., 1994). In our previous study (Piacenti et al., 2019), PNA sequences were designed using miRNA-34a structure as a model template. More specifically, the proposed PNAs had the same sequence of tumor-suppressor miRNA-34a and were able to target 3'UTR MYCN mRNA through non-perfect base pairing as miRNAs do. This hybridization fashion distinguished them from antisense class (whose members have full complementarity to the mRNA coding sequence) or miRNA-mask (which requires full complementarity to the mRNA 3'UTR). These PNA analogs of different length were tested in the “miRNA-34a–MYCN” axis (Ruiz-Perez et al., 2017), being the MYCN oncogene a validated target of tumor suppressive miRNA-34a in different cancers (Wei et al., 2008), including Neuroblastoma (Stallings et al., 2010; Zammit et al., 2018). MiRNA-34a was proven a crucial regulator of MYCN protein expression by binding two different regions of the 3'UTR on MYCN mRNA and silencing its translation to protein (Figure 1A) (Wei et al., 2008). PNA-based antisense and antigene strategies have also been employed to target MYCN (Pession et al., 2004; Tonelli et al., 2005). However, the opportunity of targeting MYCN via its connection with miRNA-34a could open up a new avenue for the development of innovative therapeutic strategies. Indeed, all the PNA analogs previously employed by our group to target both binding sites on MYCN mRNA 3'UTR showed very promising features in terms of stability, affinity and cellular uptake (Piacenti et al., 2019). As a complementary research on this model system, we have studied herein a PNA 8-mer fragment (tiny PNA1) reproducing the sole “seed region” of miRNA-34a. Our interest toward short, single-stranded PNA analogs was prompted by the consideration that the synthesis of longer PNAs (16–22 bases long) can be an expensive, and not always easy task, especially for sequences containing high cytosine and guanine content (≥50%) or where structural modifications such as long peptide carriers and/or fluorescent labels, are required. Furthermore, very short LNAs modified as phosphorothioate oligonucleotides (8 bases), able to recognize and silence a family of miRNA sharing the same seed region with negligible off target effects, were recently reported by (Obad et al., 2011).

Along with previously reported information, this study aims principally to deliver further significant structural insights to guide the design of chimeric PNA/RNA-based analogs. To achieve our goal, the portion from nucleobase 1 to 8 of miRNA-34a (corresponding to the “seed” region, positions 2-7/8 from the miRNA 5'-end Figure 1) was reproduced as a PNA 8-mer fragment (tiny PNA) and its structural properties, along with its interaction with target 3′UTR of MYCN mRNA (Figure 1) were investigated by a solid multidisciplinary approach relying on UV, Circular Dichroism, Molecular Dynamics simulations and, differently from our previous recent work (Piacenti et al., 2019) also involving NMR spectroscopy. A preliminary uptake assay on Neuroblastoma Kelly cells was carried out as well.

Thus, far only a relatively small number of NMR studies related to PNA sequences have been reported in literature. A pioneering work was published by Brown et al. (1994), describing the NMR solution structure of a PNA/RNA duplex, where the thymine backbone in the PNA monomer was enriched with ^13^C and ^15^N nuclei to allow recording heteronuclear NMR experiments and gaining more accurate knowledge about the structure and dynamics features of PNA (Brown et al., 1994). Next, 1D and 2D NMR was employed to study a palindromic 8-base pair PNA duplex in H_2_O and H_2_O/D_2_O solutions and revealed a structural organization with a P-type helix and helical features similar to those seen in the PNA crystal structures (He et al., 2008). Further NMR studies coupled to distance-restrained molecular dynamics of a γ-methylated, palindromic, PNA duplex highlighted that it assumed a P-type helical structure resembling those formed by canonical unmodified PNA sequences but provided also with some sort of A-like helical characteristics (He et al., 2010). Other interesting structural NMR analyses regarded PNA-dimers formed by chemically modified sequences containing caffeic acid and a guanine (Gaglione et al., 2013) and γ-sulfate PNA (Avitabile et al., 2012). Remarkably, in 2016 Liu et al. (2016) synthesized PNA monomers provided with diverse 5-halouracils that were able to form duplexes with complementary DNA or RNA with large affinity and characterized them also by 1D ^1^H NMR spectra (Liu et al., 2016). More recently, NMR studies were carried out on aep(aminoethylprolyl)-PNA to analyze formation of hybrid DNA:aep-PNA i-motif structures (Gade and Sharma, 2017). Finally, more complex structural organizations have also been investigated by NMR spectroscopy such as PNA/dsRNA triplexes (Kotikam et al., 2019). In addition in last decades, MD studies have proven useful for the understanding of the structural organization and the binding properties of PNA, enabling prediction on PNA molecular activity upon structural modifications for the improvement of DNA and RNA targeting (Sanders et al., 2013; Autiero et al., 2014, 2015; Verona et al., 2017; Jasiński et al., 2018; Piacenti et al., 2019).

In this context, our work further stresses out the relevance of NMR spectroscopy coupled to molecular dynamics to collect key structural information that could be employed to design potent PNA analogs targeting specific RNA sequences for therapeutic applications.

## Results and Discussion

Since their early days, PNAs were recognized as potent tools to carry genetic information on a chemically simple scaffold made of amino acid building blocks, which opens to a wider and more accessible range of possible monomer or polymer modifications compared to oligodeoxy/oligoribonucleotides. The assembly of PNA polymers can be carried out with automated synthesizers for solid-phase using Fmoc/Bhoc chemistry instead of the notably more challenging synthesis of nucleic acids, making PNAs more accessible even at a higher scale. Being not completely peptides nor natural nucleic acids, these constructs are biologically stable to enzymes (proteases, peptidases, or nucleases) as well as to acidic and basic conditions, and high temperatures. Moreover, the peptide-like backbone confers unique properties not found in other classes of nucleic acid analogs; for example, the lack of charges in the PNA backbone results in the loss of electrostatic repulsion between the PNA and RNA strands, thus improving the affinity toward its targets (Moccia et al., 2020a). Finally, PNA represents a more sequence-specific binder, able to discriminate single-point mutations better than its DNA or RNA analogs (Moccia et al., 2020b).

### Synthesis and Spectroscopic Characterization of Tiny PNA 1

**Figure 2:**
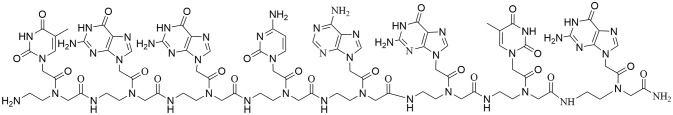
Chemical structure of the tiny PNA1.

MiR-34a seed region (PNA1) was selected as a template for the design of PNA-based analogs especially for its crucial role in target recognition (Wei et al., 2008). Given the ability of longer sequences to bind miR-34a target, as showed in our previous work, the use of such tiny fragment, once proven effective, would offer further synthetic advantages. The tiny PNA1 sequence and analogs provided with fluorescence probes were synthesized following previously established protocols (Avitabile et al., 2010; Piacenti et al., 2019) (Figure 2, Supplementary Figures 1–3 and Table 1). PNA1 (Table 1, Entry 1) was synthesized using standard solid phase peptide synthesis (SPPS) on a PAL-PEG resin and using Fmoc/Bhoc commercially available PNA monomers (Piacenti et al., 2019). PNA1 was employed for structural characterization by UV, CD and ^1^H-NMR. Similarly, in order to assess cellular uptake, an additional PNA sequence (Table 1, Entry 2) was synthesized combining PNA1 with a short carrier peptide (Piacenti et al., 2019) consisting of three positively charged lysine residues at the C-terminus (K3) and one lysine (K) at the N-terminus, an Ahx (Amino hexanoic acid) linker and FITC (Fluorescein IsoThioCyanate) as fluorescent tag (Supplementary Figures 2, 3).

**Table 1:** PNA sequences are written from N to C termini.

**Entry**	**Name**	**Sequence (N-C)**
1	PNA1	tggcagtg
2	FITC-PNA2	FITC- Ahx -K- tggcagtg—KKK-NH_2_
3	FITC-PNA3	FITC- Ahx -CK - tggcagtg—KKK-${\text{NH}}_{2}^{*}$

*Entries 1 and 2 are those directly implemented in our work*.

* *FITC-PNA3 sequence is also indicated as it was employed for comparing cellular uptake data (Piacenti et al., 2019)*.

**Figure 3:**
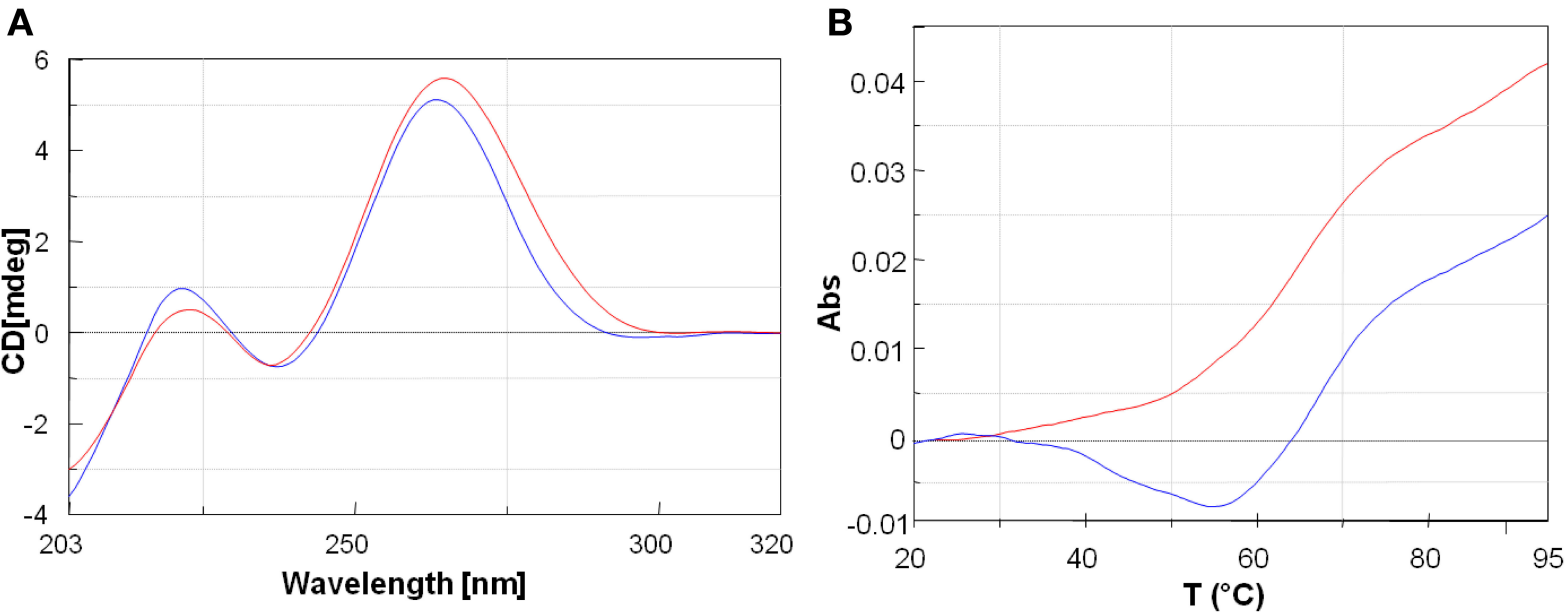
**(A)** Overlayed CD profiles of PNA1/RNA-1 (blue) and PNA1/RNA-2 (red) duplexes at 2.5 μm concentration, in 100 mM NaCl, 10 mM phosphate buffer pH = 7.4. **(B)** Overlay of UV-melting curves at 260 nm of PNA1/RNA-1 duplex T_m_ = 64°C (blue) and PNA1/RNA-2 duplex T_m_ = 60°C (red) in 100 mM NaCl, 10 mM phosphate buffer pH = 7.4.

The peptide employed as carrier in this study was also previously reported by Fabani et al., to aid the internalization of a 25-mer PNA on Huh7 cells at physiological pH (Fabani et al., 2010). The FITC-PNA3 sequence bearing one lysine and one cysteine at the N-terminus and three lysine residues at the C-terminus (K3) was reported in our previous work and herein used for comparison (Piacenti et al., 2019) (Table 1, Entry 3). The interaction between tiny PNA1 and corresponding binding regions of 3′UTR MYCN mRNA (hereafter RNA-1 and RNA-2), as well as the stability of the complexes, were assessed by CD and UV-VIS spectroscopy. The sequences of the two hetero-duplexes PNA1/RNA-1 and PNA1/RNA-2 are reported in Figure 1B. Indeed, RNA-1 and RNA-2 only differ for one base at 3' mRNA, which is a terminal position, leading to a fully complementary duplex in PNA1/RNA-1 and a mismatched one in PNA1/RNA-2. Formation of the PNA1/RNA-1 and PNA1/RNA-2 duplexes, was assessed by CD; spectra (Figure 3A) were registered for the 1:1 complexes which were annealed by a reported procedure (See Material and Methods for details). Both complexes showed the conventional shape of RNA/PNA heteroduplexes, characterized by maxima at *ca* 260–270 and 210 and minima at *ca* 240 nm, thus corroborating the ability of PNA to form a hetero-duplex with both binding sites of 3′UTR MYCN mRNAs (Corradini et al., 2012). CD spectra of ss-RNA1 and ss-RNA2 were recorded as well and are shown in Supplementary Figure 4.

UV melting experiments of both systems showed a sigmoidal profile, which was indicative of a duplex/single-strand transition (Figure 3B). The melting temperatures resulted to be respectively, T_m1_ = 64°C (PNA1/RNA-1) and T_m2_ = 60°C (PNA1/RNA-2). The ΔT_m_ = 4°C between the two systems was consistent with the small difference between the two RNA sequences, differing only for one base at 3′ mRNA. Both systems showed high thermal stability and the Tm values were comparable to the ones of full length PNA-RNA (22 unit) complex systems already reported by us (Piacenti et al., 2019). In addition, the melting temperatures of the native 8 mer long RNA-1/miRNA-34a and RNA-2/miRNA-34a duplexes, as estimated by the Oligo-Calculator software (http://biotools.nubic.northwestern.edu/OligoCalc.html), were about 26°C, lower compared to those of PNA1/RNA-1 and PNA1/RNA-2, further proving the benefit of employing PNA based analogs for miRNA targeting despite the presence of mismatches.

### NMR Characterization

**Figure 4:**
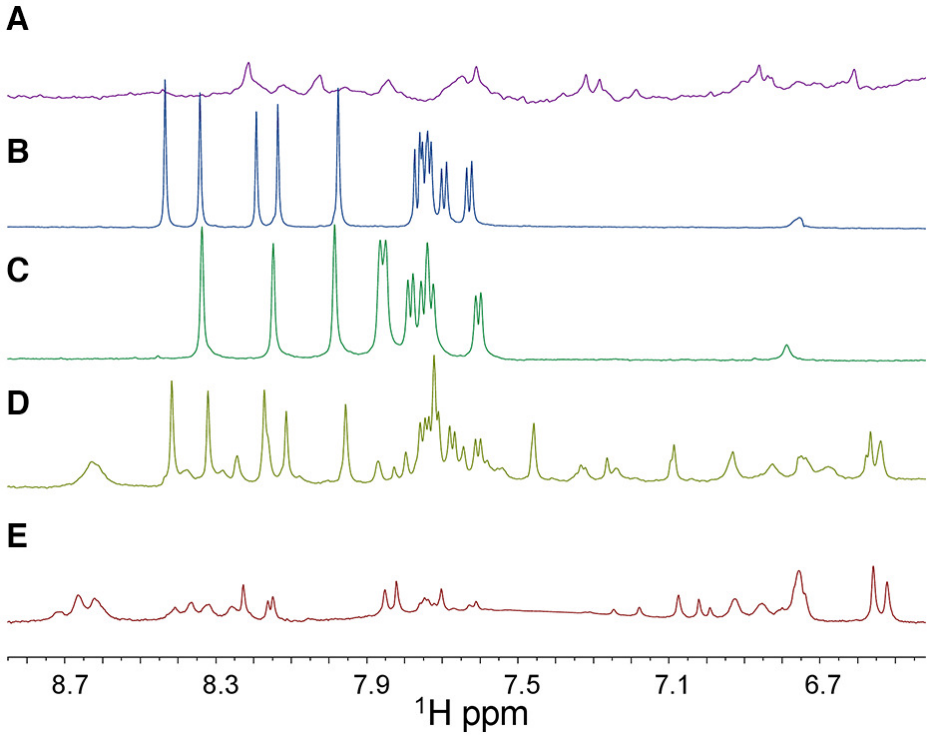
1D [^1^H] NMR spectra (only the 6.7-8.7 ppm region is shown) of ss-PNA1 **(A)**, ss-RNA-1 **(B)**, ss-RNA-2 **(C)**, PNA1/RNA-1 duplex **(D)**, PNA1/RNA-2 duplex **(E)** acquired in 10 mM NaP buffer, 100 mM NaCl, pH 7.4. In the 1D [^1^H] spectrum of the PNA1/RNA-1 duplex **(D)** a set of peaks belonging to RNA-1 in its unbound form are present due to an RNA excess in the sample. Spectra were recorded at T=299 K with 128 scans.

The 1D [^1^H] spectrum of the isolated PNA1 strand contains broad low intensity peaks (Figure 4A) which, as previously reported into the literature, are due to the slow interconversion of backbone amide bonds between cis and trans forms, allowing the PNA to assume multiple conformations (Brown et al., 1994). On the contrary, the 1D [^1^H] spectra of the ss-RNA-1 and ss-RNA-2 samples show a set of intense and sharp peaks (Figures 4B,C). Moreover, when the PNA is bound to the RNA, the backbone conformational freedom is reduced and its resonances become sharper (Figures 4D,E) (Brown et al., 1994). Chemical shifts changes with respect to isolated strands also occur when both PNA and RNA are mixed together, demonstrating that structural alterations take place upon complex formation (Figures 4D,E).

**Figure 5:**
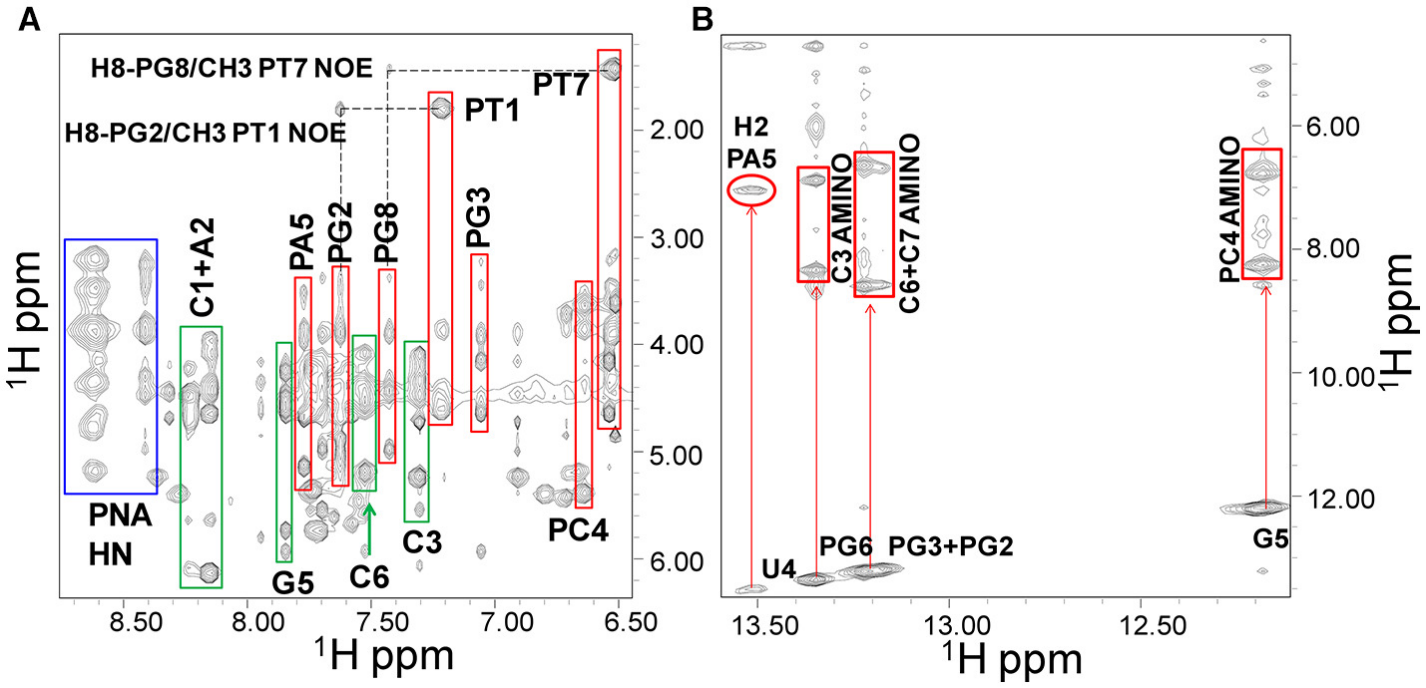
NOESY 300 spectrum of the PNA1/RNA-1 duplex in 10 mM NaP buffer, 100 mM NaCl, pH 7.4. **(A)** Expansion of the spectrum where correlations from PNA1 H_N_, RNA1, and PNA1 aromatic protons are highlighted with blue, green and red rectangles respectively. **(B)** Imino/amino-aromatic correlation region: imino/amino and imino/adenine H2 cross-peaks are indicated.

Ss-RNA-1 and ss-RNA-2 resonances were assigned by analyzing 2D [^1^H, ^1^H] NMR spectra following standard procedures (Supplementary Tables 1, 2 and Supplementary Figure 5) (Hare et al., 1983; Wuthrich, 1986). In particular, cross peaks between H5 and H6 protons of cytosine and uracile bases could be detected in TOCSY spectra (H5 protons resonating between 5.6 and 5.8 ppm and H6 around 7.6–7.8 ppm) whereas the couplings with other oxyribose protons could be identified (Supplementary Figure 6A) in the region of sugar H1' protons (5.6–6.5 ppm). Sequential assignments were achieved by analyzing NOESY spectra where the H6 protons of pyrimidines and the H8-H2 protons of purines are correlated with intra-sugar protons, along with the H1'-H2' sugar protons of the preceding nucleotide (Supplementary Figure 6B). NOE correlations between aromatic protons of adjacent bases could be detected as well. In the 2D [^1^H, ^1^H] NMR spectra of the duplexes, most PNA1 signals resonate at rather different chemical shifts compared to those belonging to the RNA and thus could be easily recognized (Figure 5A). The thymines PT1 and PT7 represented a good starting point to achieve PNA1 assignments: in 2D [^1^H, ^1^H] spectra, their methyl protons are correlated with intra-aromatic ones and, NOE correlations between methyl protons and aromatic protons of neighboring PNA residues are present (Figure 5A).

**Figure 6:**
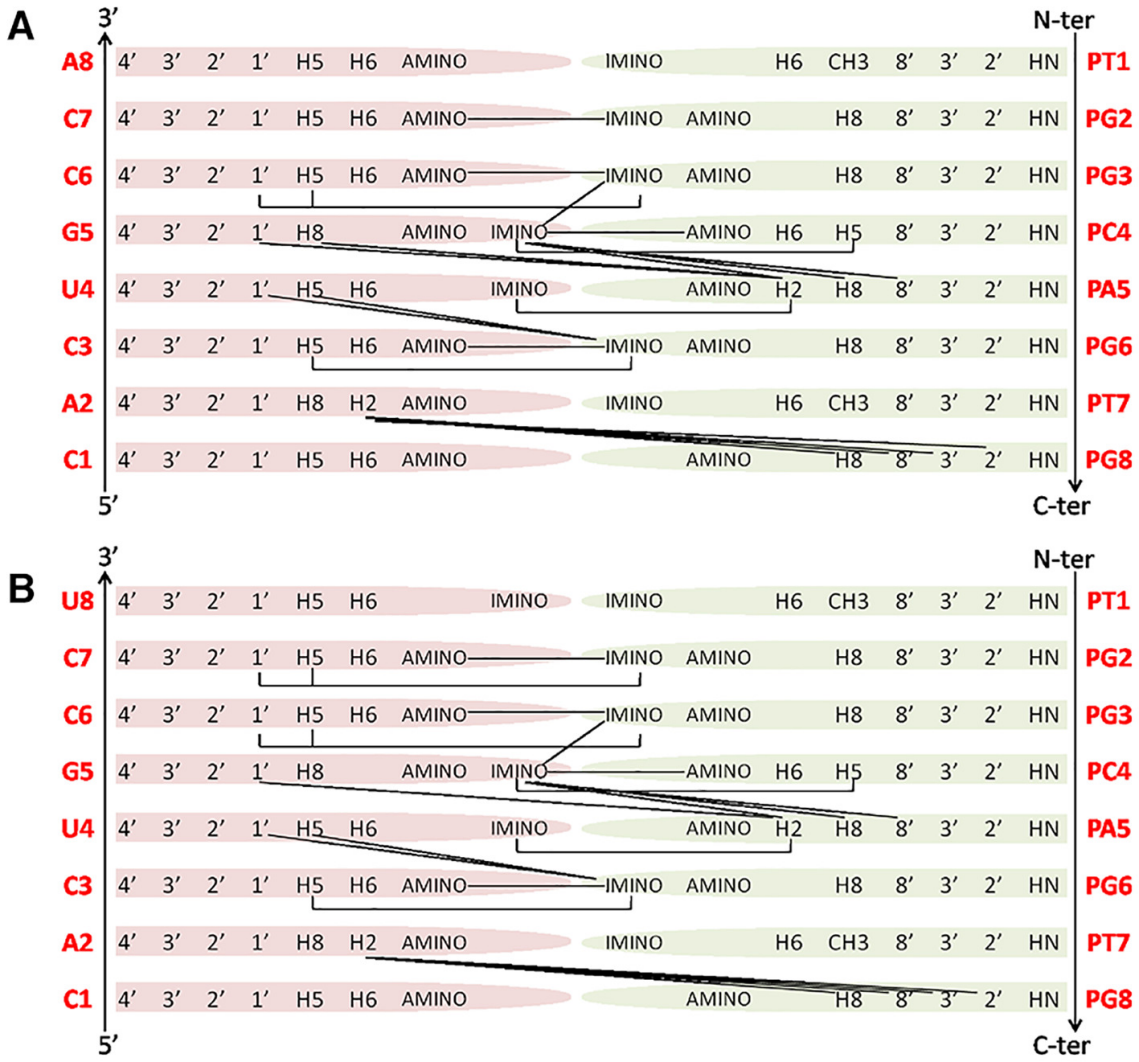
Main NOE interactions observed for the PNA1/RNA-1 **(A)** and PNA1/RNA-2 **(B)** duplexes.

Similarly to RNA, H5-H6 protons of PC4 are cross-linked in TOCSY spectra, making this residue straightforward to identify whereas, aromatic protons of flanked bases correlate with PC4 protons in NOESY spectra. Amide protons of the PNA backbone lie in the region between 8.4 and 8.7 ppm and have intra-residue NOE cross-peaks with aromatic protons (Figure 5A). Similarly, the 8' methylene protons in the PNA (Supplementary Figure 5) have correlations with aromatic protons of the same residue in NOESY spectra. Also, 3′ and 2′ methylene protons could be assigned following 8′H-3′H-2′H correlations in NOESY spectra in the region between 3.4 and 5.0 ppm, or their scalar interactions with the HN backbone protons in TOCSY spectra, although assignments resulted not straightforward due to spectral overlaps. Moreover, 5′ methylene protons, located between the tertiary nitrogen and the carbonyl group (Supplementary Figure 5), lack TOCSY or NOESY correlations with other PNA protons, and for this reason could not be unambiguously assigned (Supplementary Tables 3, 4). Several imino proton resonances could be observed in the 1D [^1^H] and 2D [^1^H, ^1^H] spectra of the duplexes, one per base pair, with the exception of the less stable end base pairs (Figures 5B, 6 and Supplementary Figures 7B,D). The NOESY spectra of the duplexes (Supplementary Figures 7, 8) show intermolecular contacts characteristic of the Watson-Crick base pairing [i.e., strong cross-peaks between the Uracile (U4) imino and the H2 protons of the partner Adenine (PA5), between the Guanines imino protons (PG2, PG3, PG6, G5) and the amino protons of the pairing Cytosines (C7, C6, C3, PC4)] (Figures 5, 6). Moreover, NOEs between the A2 H2 proton and PG8 methylene protons, and PA5 H2 proton and G5 H1' proton are present in both duplexes spectra (Figure 6) (Brown et al., 1994). RNA-1 and RNA-2 sequences differ just in the last base, which is U8 in RNA-2 and it is not complementary to PT1.

Our NMR spectra failed to identify relevant structural differences between PNA1/RNA-1 and PNA1/RNA-2 duplexes due to the absence of NOE contacts between base pairs at both terminal positions. Altogether NMR data indicated the formation of anti-parallel duplexes with a well-organized structural core including RNA segment from C7 to A2 and PNA segment PG2-PT7 and provided with flexible termini (Figure 6).

### Molecular Dynamics Study

**Figure 7:**
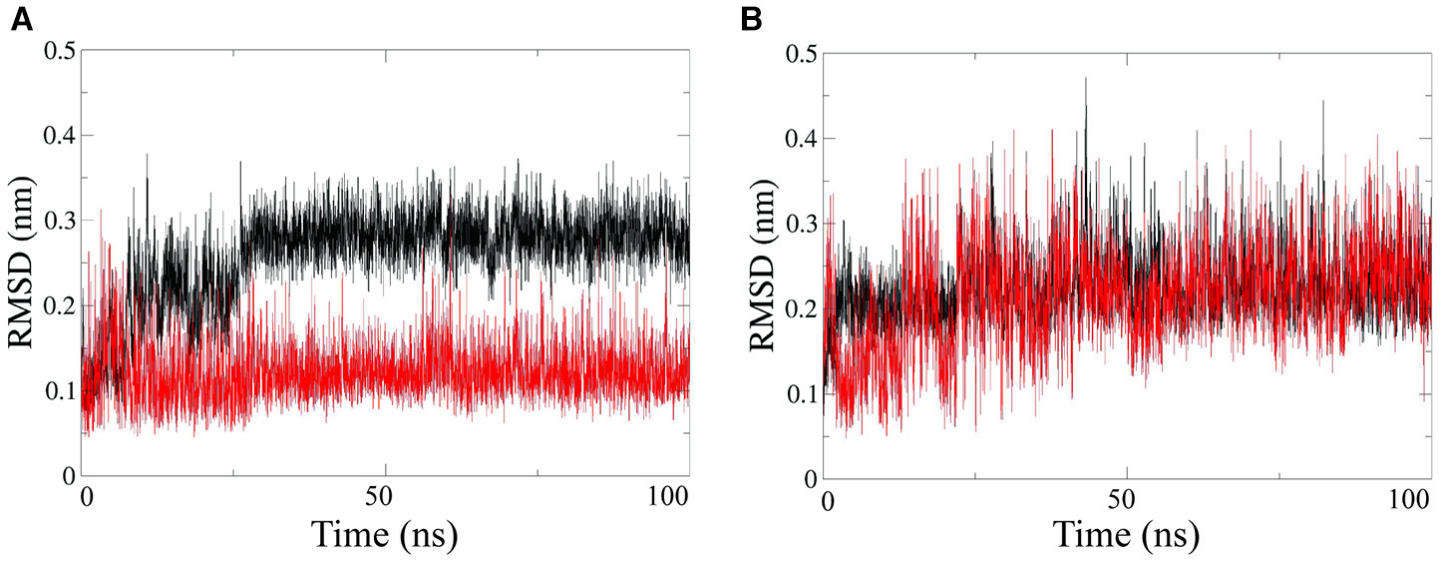
RMSD computed on PNA strand (black line) and RNA strand (red line), for PNA/RNA-1 **(A)** and PNA/RNA-2 **(B)** duplexes, respectively.

Further structural characterization of the RNA-PNA duplexes was achieved by all-atoms Molecular Dynamics (MD) simulations, performed by using the GROMACS simulation package (Van der Spoel et al., 2005; Autiero et al., 2014, 2015). MD simulations were carried out in explicit water on both PNA1/RNA-1 and PNA1/RNA-2 systems. Root Mean Square Deviations (RMSD) computed along simulations showed that each system reaches equilibrium after 10–20 ns and was stable throughout the simulation time (Figure 7). RMSD average values range from 0.12 nm to 0.22 nm for all strands except for the PNA1 strand in PNA1/RNA-1 duplex (Figure 7A), which displays a slightly higher average RMSD value of 0.3 nm.

**Figure 8:**
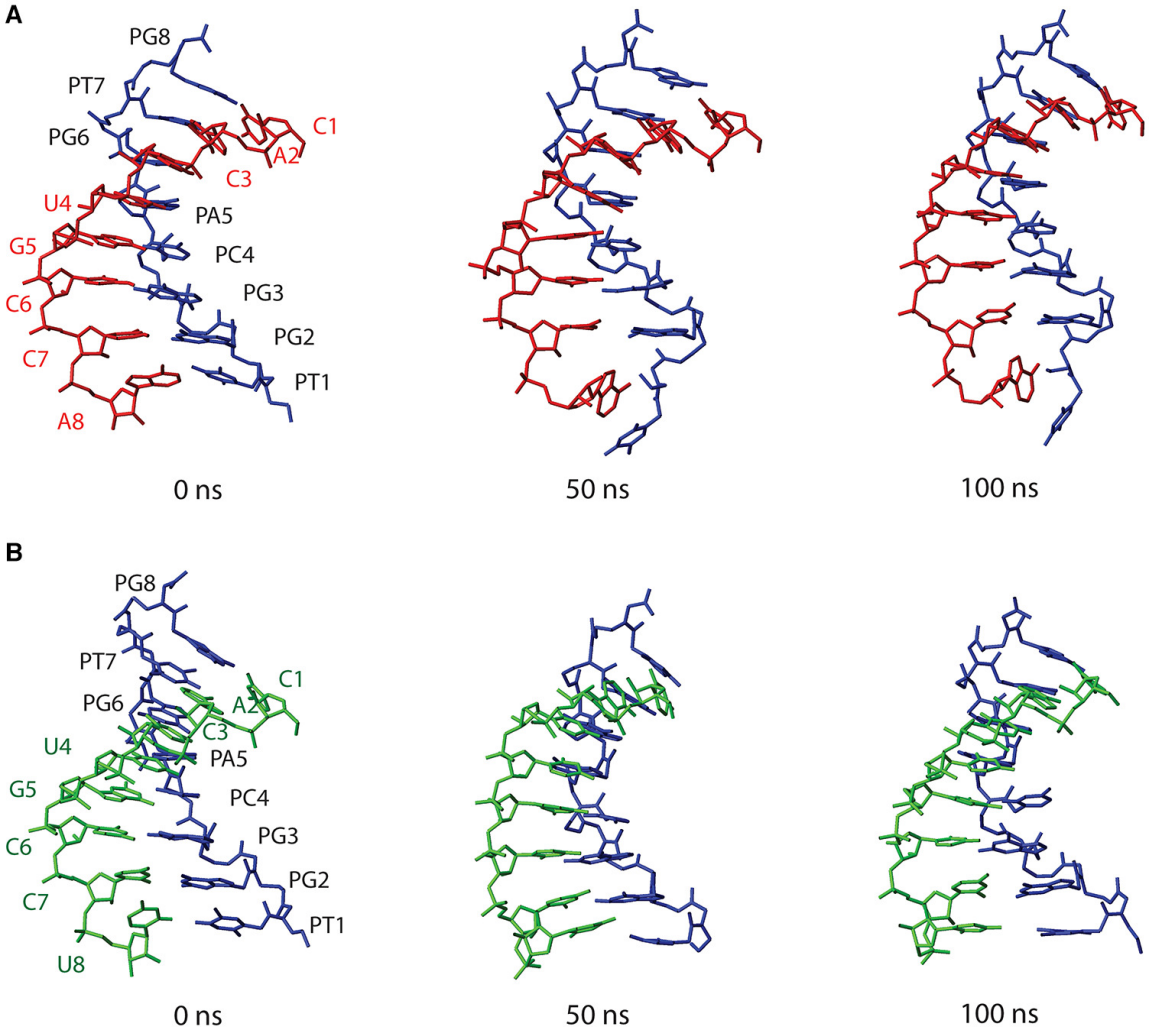
Structures of PNA1/RNA-1 **(A)** and PNA1/RNA-2 **(B)** duplexes, extrapolated from MD trajectories at different simulation times (0, 50, 100 ns). Both PNA1 and RNA strands are shown in stick representation without hydrogens, for clarity. PNA1 is displayed in blue, RNA-1 in red and RNA-2 is in green.

Figure 8 shows structures of both PNA1/RNA-1 and PNA1/RNA-2 systems, extrapolated from MD trajectories at different simulation times (0, 50, 100 ns). Base pairing and base stacking were quite well-preserved in both duplexes, albeit, local distortions from canonical geometry were present. In the case of PNA1/RNA-1, the N-terminal residue of PNA strand, namely PT1, is characterized by a high flexibility leading to PT1 base displacement, local backbone distortion and loss of PT1-A8 Watson-Crick base pairing. This finding suggests that the high RMSD value of PNA1 strand of PNA1/RNA-1 duplex, previously discussed, was due to the PT1 rearrangement. In fact, RMSD curve computed on PNA1 strand of PNA1/RNA-1 excluding the contribution of PT1 residue, shows a low average value (1.5 nm), comparable with the other strands in both PNA1/RNA-1 and PNA1/RNA-2 systems (Supplementary Figure 9). Indeed, the high flexibility of PT1 is due to the terminal position of this residue. However, this local conformational rearrangement of the terminal PT1 did not affect the stability of the overall duplex structure, as it was also assessed by the calculations of the hydrogen bonds formed between the PNA and RNA strands of the duplex during simulation time (Table 2). Indeed, data reported in Table 2 indicated that all Watson-Crick (W-C) hydrogen bonds present in PNA1/RNA-1 duplex, excluding those between PT1 and A8, had a very high percentage of existence (more than 90%). In the case of PNA1/RNA-2, PT1 residue did not undergo conformational rearrangements like in PNA1/RNA-1. However, due to the lack of complementary base on RNA-2 strand, it turns far away from U8, not being involved in Watson-Crick base pairing (Figure 8B). This finding was confirmed by the absence of PT1-U8 base pair in Table 2, indicating that these bases were not involved in stable W-C hydrogen bonds during simulation of PNA1/RNA-2.

**Table 2:** Percentage of existence (%) of hydrogen bonds made between nucleobases of the two strands (PNA and RNA) in each system along the simulation.

**PNA1/RNA-1**		**PNA1/RNA-2**	
PG2(O6) - C7(H42) ^*^ PG2(H1) - C7(N3) PG2(H22) - C7(O2) PG3(O6) - C6(H42) PG3(H1) - C6(N3) PG3(H22) - C6(O2) PC4(N3) - G5(H1) PC4(O2) - G5(H22) PC4(H42) - G5(O6) PA5(N1) - U4(H3) PA5(H62) - U4(O4) PG6(O6) - C3(H42) PG6(H1) - C3(N3) PG6(H22) - C3(O2) PT7(O4) - A2(H62) PT7(H3) - A2(N1) PG8(H1) - C1(N3) PG8(H22) - C1(O2) PG8(O6) - C1(H42)	82.5 99.2 99.1 97.1 99.7 99.6 99.5 99.6 98.5 96.5 92.4 96.4 99.5 99.4 90.5 99.6 92.9 96.6 76.9	PG2(O6) - C7(H42) PG2(H1) - C7(N3) PG2(H22) - C7(O2) PG3(O6) - C6(H42) PG3(H1) - C6(N3) PG3(H22) - C6(O2) PC4(N3) - G5(H1) PC4(O2) - G5(H22) PC4(H42) - G5(O6) PA5(H62) - U4(O2) PG6(O6) - C3(H42) PG6(H1) - C3(N3) PG6(H22) - C3(O2) PT7(O4) - A2(H62) PT7(H3) - A2(N1) PG8(H1) - C1(N3) PG8(H22) - C1(O2) PG8(O6) - C1(H42)	94.9 99.2 98.7 93.4 97.8 99.0 72.7 97.7 63.0 88.5 96.6 99.7 99.5 97.4 99.5 96.4 98.1 86.6

*Only percentages > 40% are reported*.

* *See Supplementary Figure 10 for atom nomenclature*.

Moreover, Table 2 indicated a non-canonical base pairing between PA5 and U4. Visual inspection of the PNA1/RNA-2 trajectory revealed that U4 base pointed slightly away from the duplex and PA5 was in turn rotated in order to preserve U4 base pairing, thus leading to the formation of a new hydrogen bond involving H62 and O2 atoms of PA5 and U4, respectively (Figure 8 and Table 2). The newly formed hydrogen bond was stable throughout the entire simulation (88.5% of existence), and base stacking was preserved as well. It is worth noting that this local distortion did not affect the overall duplex structure of PNA1/RNA-2. All these findings highlighted the flexibility of PNA, which is able to accommodate local structural rearrangements.

To summarize, both systems herein studied retained the overall duplex structure along the simulation. Despite the presence of the mismatch in PNA1/RNA-2, MD simulations did not reveal significant differences between the two duplexes in terms of the overall conformational stability. This can be ascribed to the mismatch being located at the terminal end of the duplex (3' end of RNA strands) which is inherently flexible in both duplexes.

### Preliminary *in vitro* Assay in Kelly Neuroblastoma Cells

**Figure 9:**
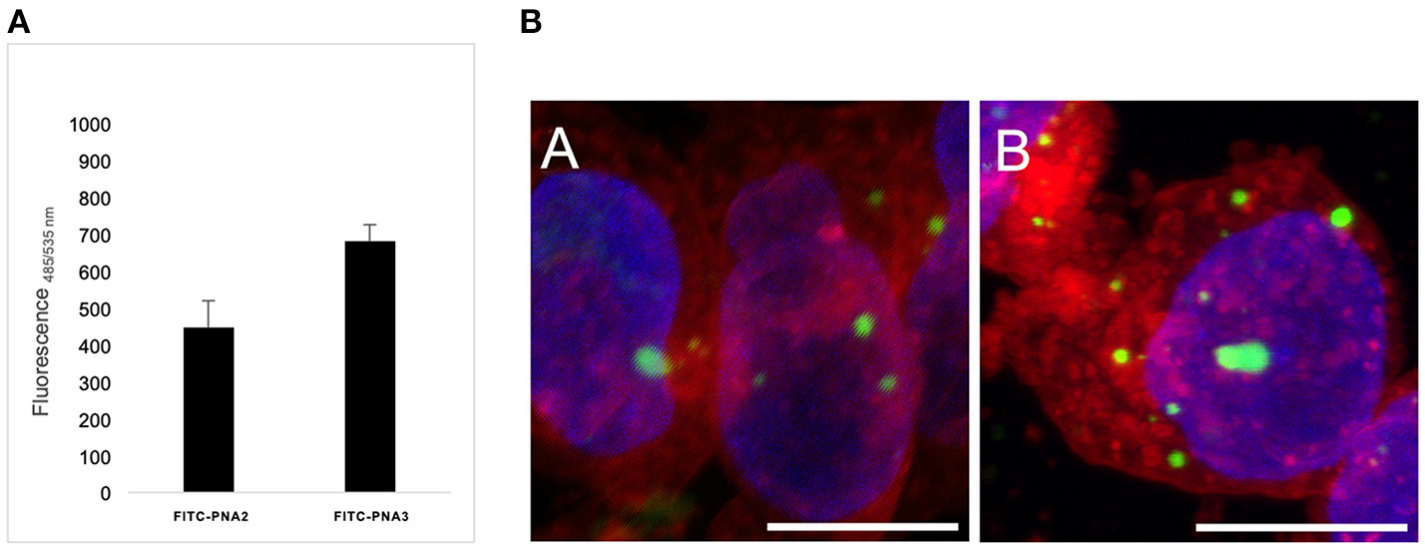
**(A)** Cellular intake of FITC-PNA2 vs. FITC-PNA3 at 0.1 μM concentration. All experiments were performed on Kelly cells in 3 biological repeats and 4 technical repeats. **(B)** Confocal microscopy of Kelly cells treated with 2.5 μM solution of (a) FITC-PNA2; (b) FITC-PNA3. Green, FITC; red, Cell Mask TM Orange membrane staining; blue, Hoechst DNA staining. Scale bar corresponds to 10 μm in all images. All images were taken at the same confocal microscopy setting.

Cellular uptake of the described tiny PNA sequence was studied *in vitro* on Human Kelly neuroblastoma cell line, without the use of any transfection agents, and compared to previously reported results from our group (Piacenti et al., 2019). In particular, the PNA oligomers were provided with short peptide carriers at both termini consisting of a trilysines segment at the C-terminus and one lysine at the N-terminus (FITC-PNA2) or Cys-Lys dipeptide at the N-terminus (FITC-PNA3) (Table 1), that was previously reported by Fabani et al. (2010) to promote cellular uptake for PNA single strands. Kelly cells were incubated for 24 h with FITC-PNA2 or FITC-PNA3 sequences individually, at minimal 0.1 μM concentration in optiMEM, without the use of any transfecting agent and using a protocol for miRNA mimics delivery, which was previously optimized (Curtin, 2018). Both peptide carriers were able to provide internalization of PNA sequences albeit to a different extent (Figure 9A); uptake was slightly higher than 3% for FITC-PNA2 and almost 5% for FITC-PNA3.

The FITC-PNA2 and FITC-PNA3 cellular uptake was also assessed by confocal microscopy (Figure 9B). The membrane/nuclei optimal staining protocol and stain compatibility were screened on live and fixed cells in order to achieve the best image resolution. The coverslips were pre-treated with collagen prior to cells being seeded and incubated for 24 h with 2.5 μM solutions for each of PNAs. In both cases the fluorescence emitted from inside the cell was localized in vesicular shapes in the cytosol. This observation confirmed, as previously reported by Torres et al. with similar constructs, that PNAs conjugated to cationic and thiol-containing carriers were transported inside the cell by different endocytic mechanisms, hence leading to their incorporation into endosomes (Fabani et al., 2010). The cellular uptake of tiny FITC-PNA2 and FITC-PNA3 in the absence of any transfection agent was unexpectedly low if compared to longer sequences, as previously reported by our group (Piacenti et al., 2019). The employment of endosome disrupting agents and dose/uptake correlation needs further exploration as well as conjugation to different peptide carriers (R8) or different delivery strategies (e.g., liposomes).

## Conclusion

Our group has recently reported a study on the employment of PNA-based analogs of tumor suppressor miRNA-34a, which showed promising features in terms of stability and cellular uptake, for the targeting of MYCN. In the present research, we studied through a combined approach consisting of computational and experimental studies that a tiny PNA (8-mer), perfectly matching with region 1–8 of miRNA-34a (corresponding to the “seed” region), is able to recognize its target on the 3'UTR of MYCN mRNA. The sequences of the two heteroduplexes PNA1/RNA-1, and PNA1/RNA-2 studied in this work, only differ for a single base at 3' mRNA, which is at terminal position, leading to a fully complementary duplex in PNA1/RNA-1 and a mismatch in PNA1/RNA-2. NMR data indicated the formation of anti-parallel duplexes with a well-organized structural core including the RNA segment from C7 to A2 and the PNA segment PT2-PG7 provided with flexible termini. Further structural investigation by MD simulations evidenced that base pairing and base stacking were maintained in both hetero complexes, thus preserving the overall duplex structure throughout the simulations, in agreement with the organized structural core highlighted by NMR studies. Furthermore, consistently with NMR data, a significant flexibility of the N terminal part (PT1 base) of both duplexes emerged from MD simulations. UV and CD spectroscopic data confirmed the ability of PNA1 to bind both 3′UTR MYCN mRNA binding sites forming stable PNA/RNA complexes. The predicted Tm values of correspondent natural RNA-miRNA-34a complex lied in a much lower range if compared to results obtained in this work for PNA/RNA complexes. Preliminary *in vitro* assays were also carried out showing low PNA internalization, which needs to be further investigated. All these findings, consistently with the higher affinity of PNAs over RNAs toward complementary RNA strand, strengthen the interest toward the use of peptide nucleic acids as effective miRNA targeting agents. In particular, we believe that PNAs represent a perfect tool in drug discovery to develop PNA-based or PNA-RNA chimeric miRNA analogs, due to their superior physico-chemical properties and to a number of synthetic advantages compared to RNA/DNA in terms of cost/time-saving, especially if shorter sequences can be implemented. With the present study we wish to shed light on the structural insights to guide the design of chimeric PNA/RNA sequences, elucidate minimal filaments length required to retain function and fine-tuned physico-chemical properties. Once fully optimized, such approach would provide a significant platform to design analogs of any tumor suppressor miRNAs.

## Materials and Methods

### General Information

PNA monomers (Fmoc-A(Bhoc)-OH, Fmoc-T-OH, Fmoc-G(Bhoc)-OH and Fmoc-C(Bhoc)-OH) were purchased from Link Technologies, 2-(1H-7-Azabenzotriazol-1-yl)-1,1,3,3-tetramethyluronoium hexafluorphosphate (HATU) was purchased from IRIS Biotech GMBH, the Fmoc-PAL-PEG-PS resin was purchased from Applied Biosystem. Acetonitrile (ACN) was from Romil, dry N,N-dimethylformamide (DMF), Triisopropylsilane (TIS), 2,6-Lutidine and all other reagents were from Sigma Aldrich (MERCK). LC–MS analysis was performed on an LC–MS Agilent Technologies 6230 ESI-TOF instrument with a Phenomenex Jupiter 3 mm C18 (150_2.0 mm) column at a flow rate of 0.2 mL/min. Purification was carried out on a Phenomenex Jupiter 10 μ Proteo 90 Å (250 × 10 mm) column. Purification was carried out by RP-HPLC with a Shimadzu LC-8A, equipped with a SPD-M10 AV diode array detector using a Kinetex® 5 μm C18 100 Å, AXIA Packed LC Column 50 × 21.2 mm, a column with a flow rate of 20 mL min^−1^. PNA oligomers were obtained with a purity of >95%; yields were calculated based on the amount of pure PNAs obtained after purification.

### PNA Synthesis

PNA1 oligomer was synthesized on a 2 μmol scale using the standard PNA protocol. The synthesis was performed on a Fmoc-PAL-PEG-PS resin (0.16 mmol/g). To improve the coupling efficiency double couplings were carried out on PNA-guanine and adenine monomers. Synthesis was carried out using repetitive cycles of deprotection, coupling and capping at room temperature. After each of these cycles two flow washes (25s) with DMF were performed. Deprotection: 20% piperidine in DMF, 7 min. Coupling: 5 equivalents of PNA monomers were dissolved in DMF to a concentration of 0.22M; a solution of HATU in DMF 0.18M (4eq) and 50 μL of a mixture of DIPEA 0.2 M and 2,6-Lutidine 0.3 M in DMF were added (Pre-activation time: 1 min- coupling time: 20 min). Capping: acetic anhydride/2,6 Lutidine/DMF (5/6/89 v/v/v/), 5 min. At the end of the synthesis the resin was washed with DMF, DCM, diethyl ether and dried *in vacuo*. The PNA oligomer was cleaved from the resin and de-protected by a treatment with a solution of TFA/H_2_O/TIS (95/2.5/2.5 v/v/v) for 90 min at room temperature. The filtrate was flushed by a stream of nitrogen to remove the most of TFA and the PNA was precipitated by addition of cold diethyl ether, washed three times with diethyl ether and dried *in vacuo*. Purification was carried out by semi-preparative RP-HPLC applying a linear gradient of acetonitrile (0.1% TFA) from 5 to 50% in 20 min. PNA purity and identity were confirmed by LC-MS system as previously described: a gradient of solvent B (0.05% TFA in CH_3_CN) from solvent A (0.05% TFA in H_2_O) of 5% to 50% was applied over 20 min. PNA was identified by electrospray mass analysis. Calculated: 2240.6012; [M+2H]^2+^: 1121.3006; [M+3H]^3+^: 747.8670; [M+4H]^4+^: 561.1503; Found: 2240.9301; [M+2H]^2+^: 1121.4703; [M+3H]^3+^: 747.9841; [M+4H]^4+^: 561.2416. FITC-PNA2 synthesis: The synthesis of FITC- PNA2 sequence was carried out in solid phase see reference (Piacenti et al., 2019) for the complete synthetic and purification procedure. The identity of the product was confirmed by MALDI system using α-cyano-4-hydroxycinnamic acid as a Matrix (10 mg/mL in 50:50 water/acetonitrile (0.1% TFA final concentration). MALDI-TOF C_138_H_179_N_59_O_35_S (m/z): Found 3255.5515 required: 3255.3829 for [M+H]^+^.

### UV Melting Experiments and CD

UV measurements were carried out on a JASCO V-550 UV/VIS spectrophotometer equipped with a Peltier block ETC-505T temperature controller by using 1 cm quartz cells of both 0.5 and 1 mL internal volume (Hellma). Single strand PNA and complementary RNA were dissolved in ultrapure water and were annealed by warming up at 90°C and slowly cooling down to 4°C in Phosphate buffer 10 mM, 100 mM NaCl, pH = 7.4. The thermal denaturation experiment was carried out using 2.5 μM duplex in Phosphate buffer 5 mM, 50 mM NaCl, pH = 7.4 at a 0.5°C/min scan speed, recording at 260 nm in duplicate. CD spectra were recorded on a Jasco-715 spectrophotometer. CD spectra were recorded at 20°C using a 1 cm quartz cell, a 320–210 nm measurement range, 50 nm/min scanning speed, 1 nm bandwidth, 4 s response time, 2.0 nm data pitch and are the results of 3 scans.

### NMR Studies

NMR experiments were acquired for the following samples: the single strand (ss) PNA at 362 μM concentration, the ss-RNA-1 at 600 μM concentration, the ss-RNA-2 at 357 μM concentration, the PNA/RNA-1 duplex (550 μM PNA/600 μM RNA-1), and the PNA/RNA-2 duplex (335 μM PNA/335 μM RNA-2). The analyzed PNA and RNA sequences are reported in Table 3.

**Table 3:** PNA and RNA sequences analyzed by NMR experiments.

PNA1	N tggcagtg C
RNA-1	5^′^-CACUGCCA-3^′^
RNA-2	5^′^-CACUGCCU-3^′^

All the samples were dissolved in 10 mM sodium phosphate buffer at pH 7.4 with 100 mM NaCl and 10% D_2_O (98% D, Armar Scientific, Switzerland). To assign non-exchangeable protons, the 8-mer duplexes were also lyophilized, dissolved in 98% D_2_O and additional NMR experiments were conducted. 1D [^1^H] and 2D [^1^H,^1^H] TOCSY (Total Correlation Spectroscopy) (Griesinger et al., 1988), NOESY (Nuclear Overhauser Enhancement Spectroscopy) (Kumar et al., 1980), and ROESY (Rotating frame Overhauser Enhancement Spectroscopy) (Bax and Davis, 1985) experiments were collected with 16-64 scans, 128-256 FIDs in t1, 1024 or 2048 data points in t2 at a temperature of 299 K, on a Varian Unity Inova 600 MHz spectrometer provided with a coldprobe. TOCSY experiments were recorded with 70 ms mixing time, NOESY experiments with 200 and 300 ms mixing times, ROESY spectra with 250 ms mixing time. Excitation Sculpting was used for water suppression (Hwang and Shaka, 1995). Spectra were processed with VNMRJ (Varian by Agilent Technologies, Italy) and analyzed with the software NEASY (Bartels et al., 1995) [contained in CARA (http://www.nmr.ch/)]. Chemical shifts were referenced to the water peak at 4.75 ppm.

### Molecular Dynamics

MC-Fold-MC-Sym pipeline (Parisien and Major, 2008) was used to obtain the three-dimensional model of the duplexes made by residues 1–8 of miRNA34a (corresponding to “seed region”) with MYCN binding site 1 (RNA-1) and binding site 2 (RNA-2), respectively. Subsequently, miRNA34a backbone was substituted with the standard *aeg*-PNA backbone, thus obtaining the starting models of PNA1/RNA-1 and PNA1/RNA-2. The two systems were subjected to molecular dynamics (MD) simulations using the GROMACS simulation package (Van der Spoel et al., 2005). The Parmbsc0 (Hornak et al., 2006) force field, a refinement of the AMBER parm9940 force field for nucleic acids (Perez et al., 2007), was employed for the simulations. Force field parameters derived for PNA from our previous work, were used (Autiero et al., 2014, 2015). The systems were centered in an octahedral periodic cell with at least 11 Å distance to the border. The cell was then filled with TIP3P water molecules and neutralized adding counter-ions. The simulations were run under NPT conditions (300 K, 1 bar) with the Berendsen coupling algorithm (Berendsen et al., 1984). All bond lengths were held fixed using the LINCS algorithm (Hess et al., 1997). The particle mesh Ewald method was applied to treat electrostatic interactions and a non-bonded cut off of 1.4 nm was used for the Lennard-Jones potential (Darden et al., 1993, 1999). Water molecules were relaxed by energy minimization and followed by 10 ps of simulations at 300 K, restraining the PNA and RNA atomic positions with a harmonic potential. Then, the systems were heated up gradually to 300 K in a five step process, starting from 50 K. After this step, the systems were simulated in NPT standard conditions for 50 ns without restraints. The analysis and visualization of the MD trajectories were carried out using GROMACS tools and MOLMOL package (Koradi et al., 1996).

### PNA Cellular Uptake Assay

Kelly cells were seeded in 6-well plates in duplicates at 0.5 × 10^6^ density. After 24 h from incubation, the growth media was replaced with serum-free media (1 mL) and cells were treated with 0.5 mL of 0.1 μM solutions of FITC-PNA2 and FITC-PNA3 in Opti-MEM. Wells were gently agitated to provide an even treatment of the cells and incubated at 37°C for 6 h. After incubation, 500 μl of 40% Fetal Bovine Serum (FBS) (Gibco) in RPMI was added to each well. Growth media was removed and wells were incubated at 37°C for 48 h and washed with 1 mL PBS. Passive Lysis Buffer (Promega) (0.5 mL) was added to each well. The plates were wrapped in tinfoil and placed on the rocker for 20 min. The cell lysates were re-suspended and plated in four replicates on a 96 well-plate. The fluorescence of the cell lysate and of the media was read in a dual beam plate reader, at 485/535 nm.

### Confocal Microscopy

Eight well-coverslips were treated with 0.2 mM solution of collagen in DMEM, and incubated at 0–4°C overnight. The wells were washed with PBS before seeding. Kelly cells were plated at 10^4^ cells/well density. After 48 h from seeding, the growth media was replaced with serum-free media and cells were treated with 200 μL of 2.5 μM solutions of FITC-PNA2 and FITC-PNA3 in Opti-MEM. Wells were gently agitated to provide an even treatment of the cells and incubated at 37°C for 6 h. After incubation, 100 μl of 40% FBS in RPMI were added to each well in the 8-well coverslip. After 96 h, the cells were gently washed once with PBS, and then treated with 1.5X solution of CellMask™ Plasma Membrane Stains (Thermo Fisher Scientific) in PBS and incubated for 10 min. After this time, the cells were washed twice with PBS, and treated for 10 min with 3.7% EM-grade paraformaldehyde, in PBS, at room temperature. The cells were washed twice with PBS and coverslipped with Vecta shield DAPI mounting medium to stain nuclei. The cells were imaged with a Carl Zeiss, LSM 710 confocal Microscope, with images presented as maximum intensity Z projections, to allow for the 2D display of the data. All images were prepared using the FIJI software (Schindelin et al., 2012), or the FigureJ Plugin (Mutterer and Zinck, 2013).

## Data Availability Statement

The original contributions presented in the study are included in the article/Supplementary Materials, further inquiries can be directed to the corresponding author/s.

## Author Contributions

MM project conceptualization, experimental design, chemical syntheses and characterization, project funding, and manuscript draft. FM and ML performed NMR studies. EL conducted MD investigation. VP and MA performed synthesis, cellular experiments. MS Project funding and manuscript draft. All authors contributed to the draft of the manuscript and approved the final version. All authors contributed to the article and approved the submitted version.

## Conflict of Interest

The authors declare that the research was conducted in the absence of any commercial or financial relationships that could be construed as a potential conflict of interest.
