# Supplementary material for: Structural Insights on Tiny Peptide Nucleic Acid (PNA) Analogues of miRNA-34a: An in silico and Experimental Integrated Approach
Source: Front Chem. 2020 Nov 23;8:568575. doi: 10.3389/fchem.2020.568575 (PMC7719796; doi:10.3389/fchem.2020.568575)

Supplementary Material

Structural insights on tiny peptide nucleic acid (PNA) analogues of miRNA-34a: an *in silico* and experimental integrated approach.

Maria Moccia, [1]* Flavia Anna Mercurio,[2] Emma Langella,[2] Valerio Piacenti,[3] Marilisa Leone,[2] Mauro F. A. Adamo[3] and Michele Saviano.[1]*

[1]Institute of Crystallography, National Research Council, Department of Chemical Sciences and Materials Technologies, via G. Amendola 122/O, 70126, Bari, Italy

[2] Institute of Biostructures and Bioimaging, National Research Council, via Mezzocannone 16, 80134 Naples, Italy

[3] RCSI, Dept. of Pharmaceutical & Medicinal Chemistry, 123 St Stephen's Green Dublin 2, Ireland.

* Correspondence addressed to

maria.moccia@cnr.it; michele.saviano@cnr.it

TABLE OF CONTENTS

Figure S1-HPLC and ESI-MS PNA1 .... pag. 2

Figure S2-HPLC trace FITC-PNA2 .... pag. 3

Figure S3-MALDI-TOF FITC-PNA2 .... pag. 4

Figure S4-CD ss RNA-1 and RNA-2 .... pag. 5

Tables S1-S4 and ***Figures S5-S8*** NMR Chemical Shift Tables
and supportive Figures .... pag. 6-13

Figures S9-S10 MD Supplementary Figures .... pag. 14-15

Figure S1. HPLC and ESI-MS PNA-1

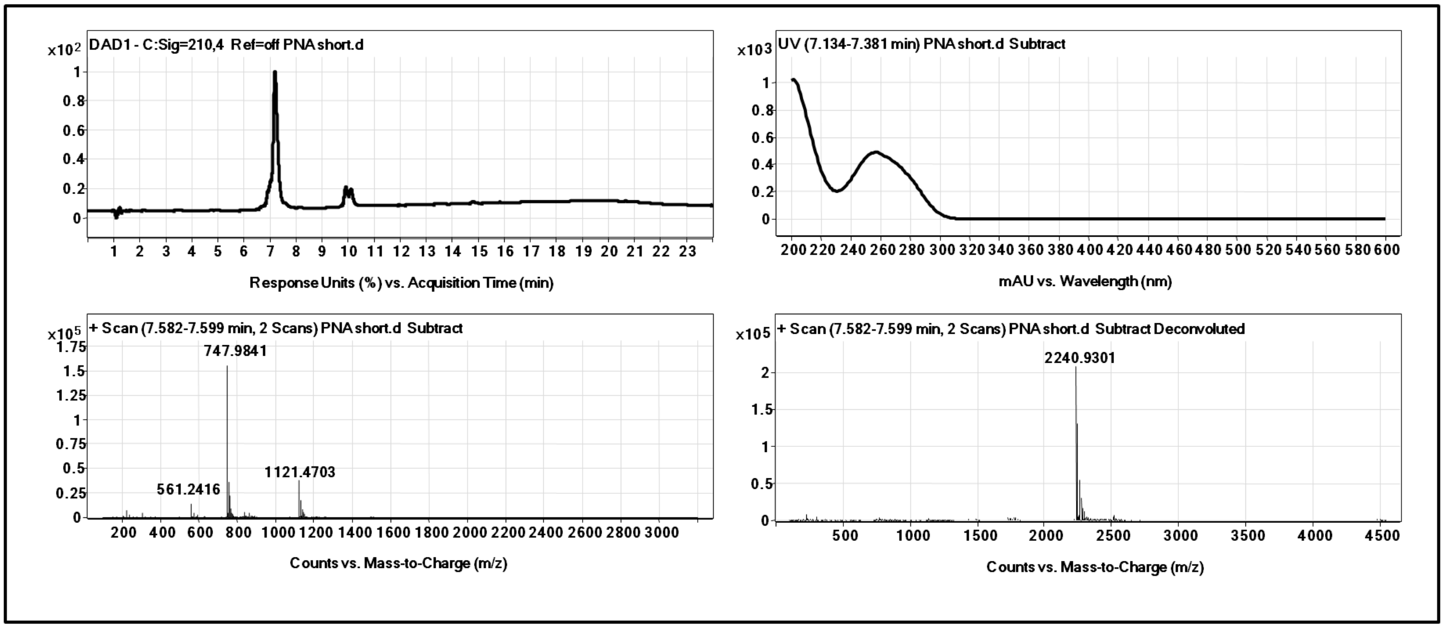

Figure S2. HPLC trace FITC-PNA2

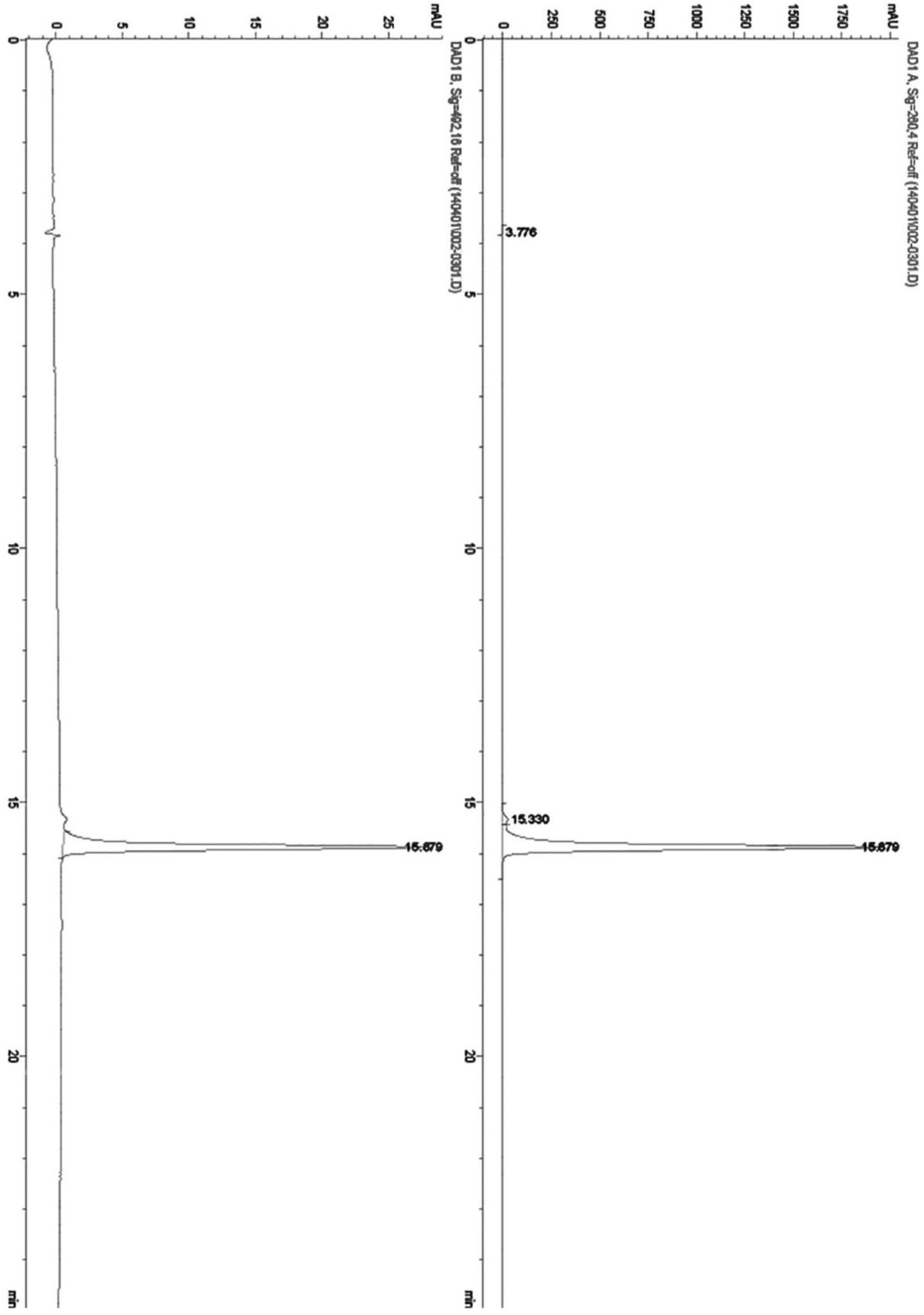

Figure S3. MALDI FITC-PNA2

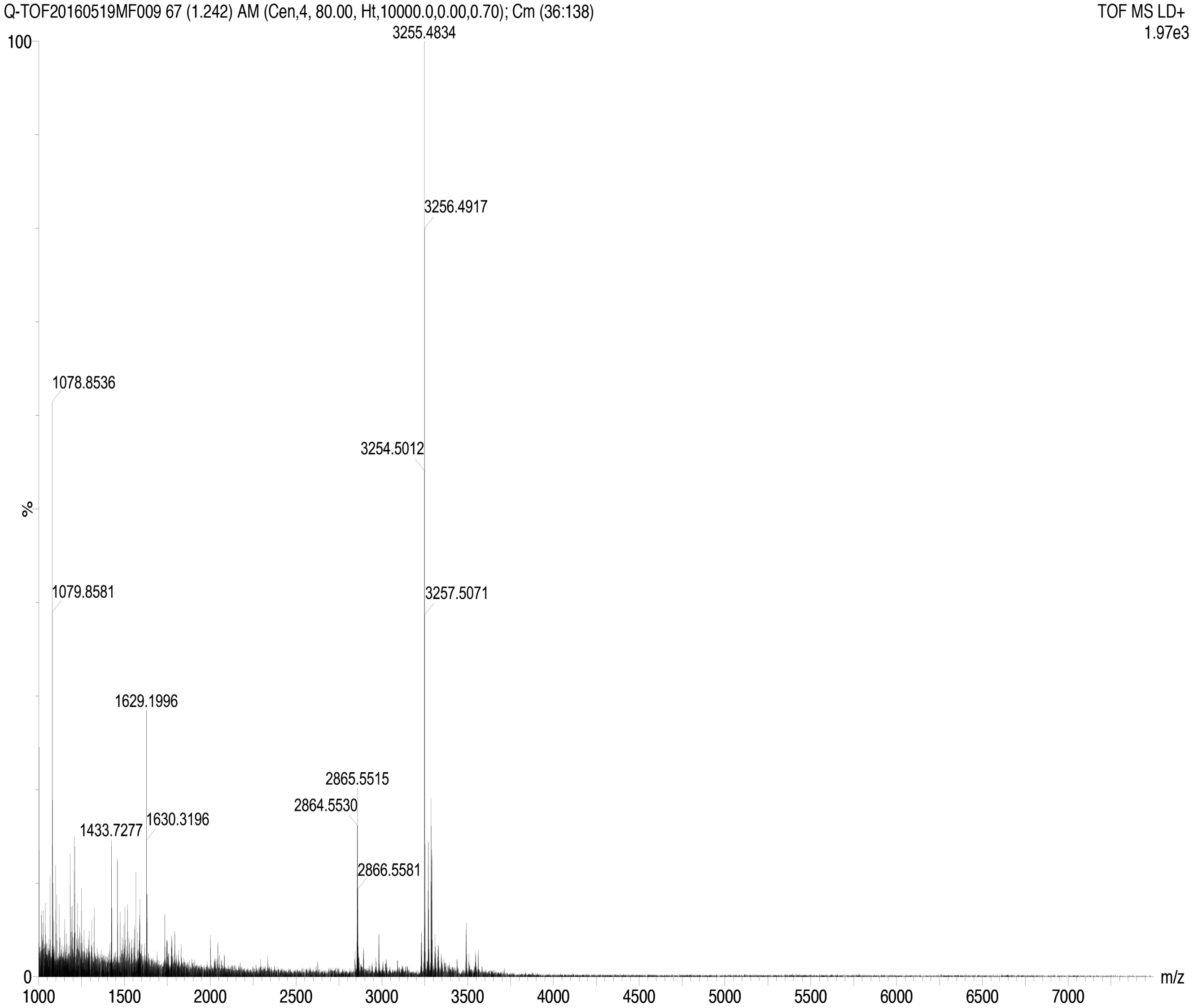

Figure S4A. CD SS RNA1 2.5 µM concentration, in 100 mM NaCl, 10 mM phosphate buffer pH=7.4.

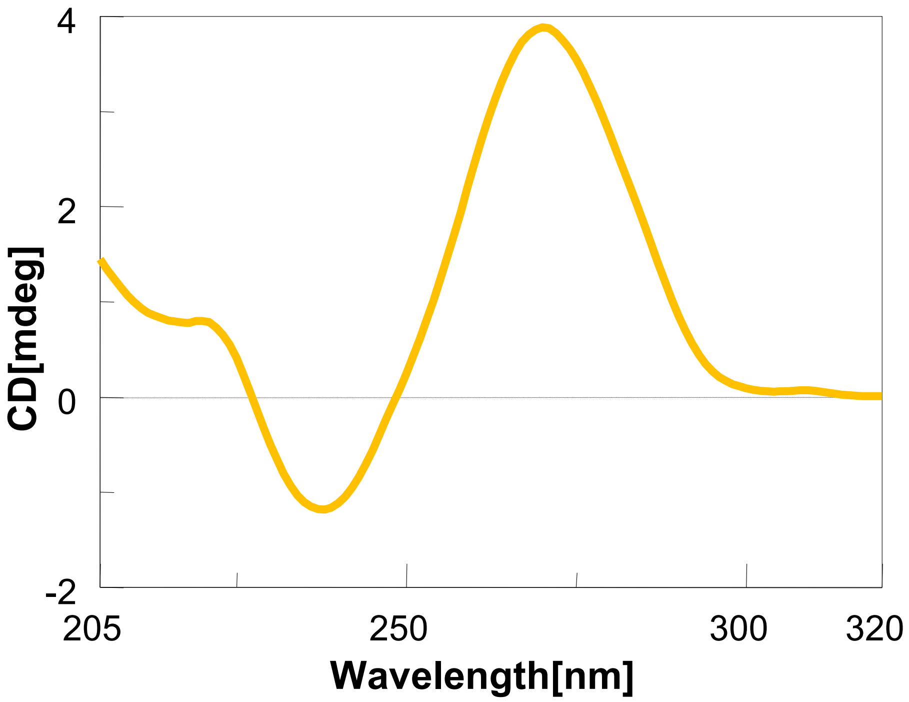

Figure S4B. CD SS RNA2 2.5 µM concentration, in 100 mM NaCl, 10 mM phosphate buffer pH=7.4.

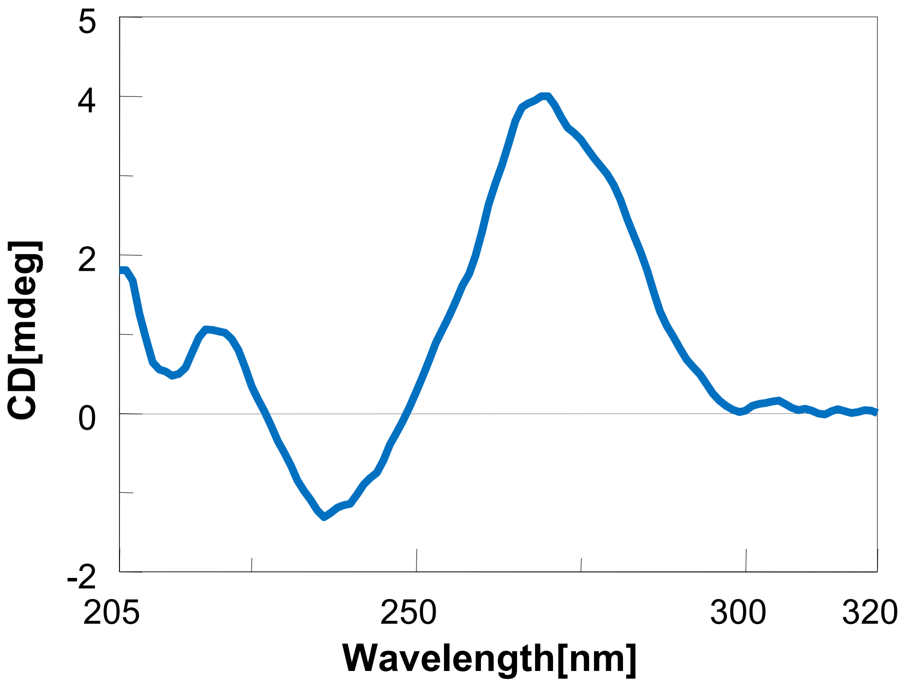

NMR

Table S1. ^{1}H chemical shifts (ppm) of ss-RNA-1 (unambiguous assignments).

	H6/H8	H2/H5	H1’	H2’	H3’	H4’	H5’/H5”
C1	7.76	5.72	5.68	4.44	4.46	4.27	3.95,3.85
A2	8.33		5.99	4.70	4.52		
C3	7.62	5.68	5.65	4.23	4.49		
U4	7.73	5.73	5.82	4.34	4.58		
G5	7.97		5.78	4.81	4.68		
C6	7.69	5.60	5.71	4.32	4.43	4.14	
C7	7.72	5.73	5.76	4.27			
A8	8.43		6.07	4.50			

Table S2. ^{1}H chemical shifts (ppm) of ss-RNA-2 (unambiguous assignments).

	H6/H8	H2/H5	H1’	H2’	H3’	H4’	H5’/H5”
C1	7.75	5.66	5.65	4.42	4.37	4.22	3.93,3.84
A2	8.30		5.97	4.65	4.52		
C3	7.57	5.62	5.58	4.17	4.45		
U4	7.72	5.70	5.79	4.32	4.54		
G5	7.95		5.76	4.78	4.66		
C6	7.70	5.61	5.69	4.34	4.45	4.13	
C7	7.84	5.73	5.77	4.28			
U8	7.82	5.74	5.90				

Figure S5. PNA and RNA protons nomenclature used in chemical shift tables.

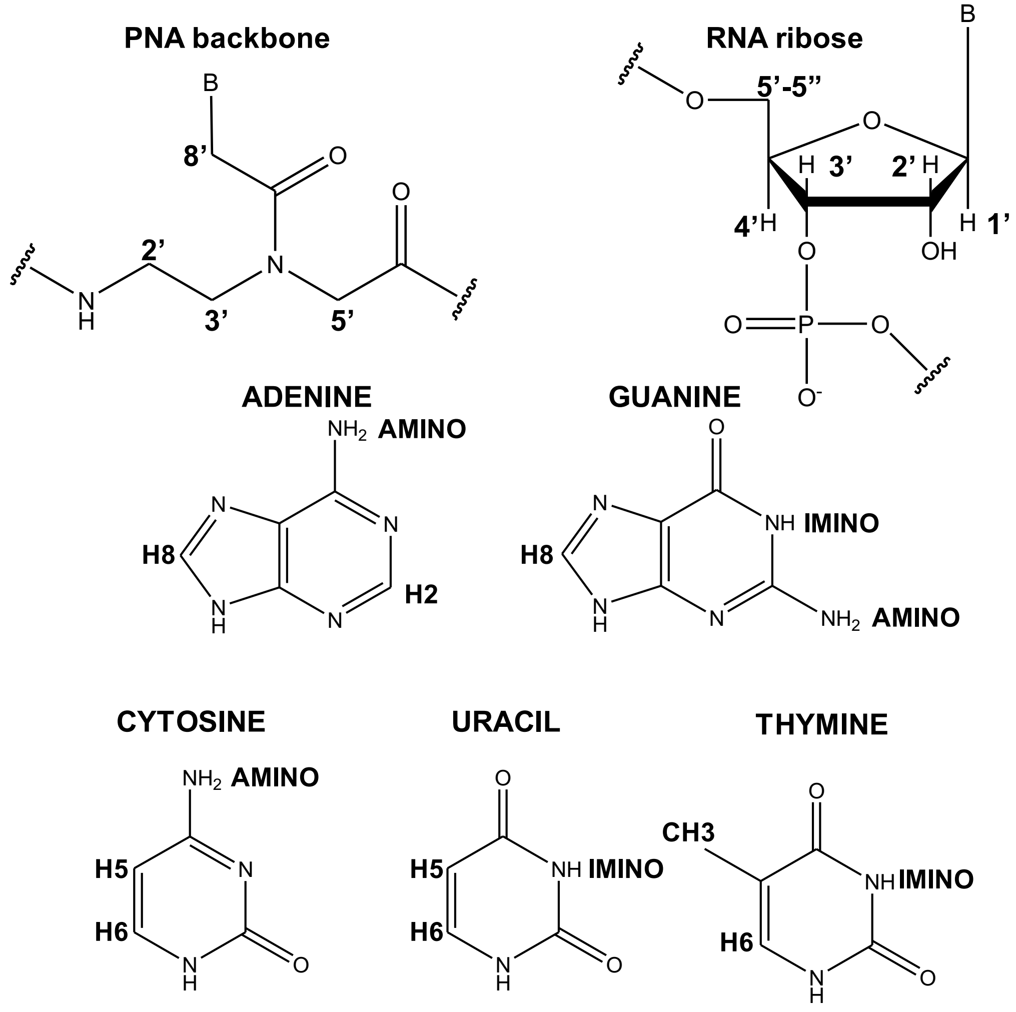

Figure S6. Spectral regions of (A) TOCSY showing the sugar H1'/pyrimidine H6 correlations and (B) NOESY containing aromatic proton correlations of ss-RNA-1.

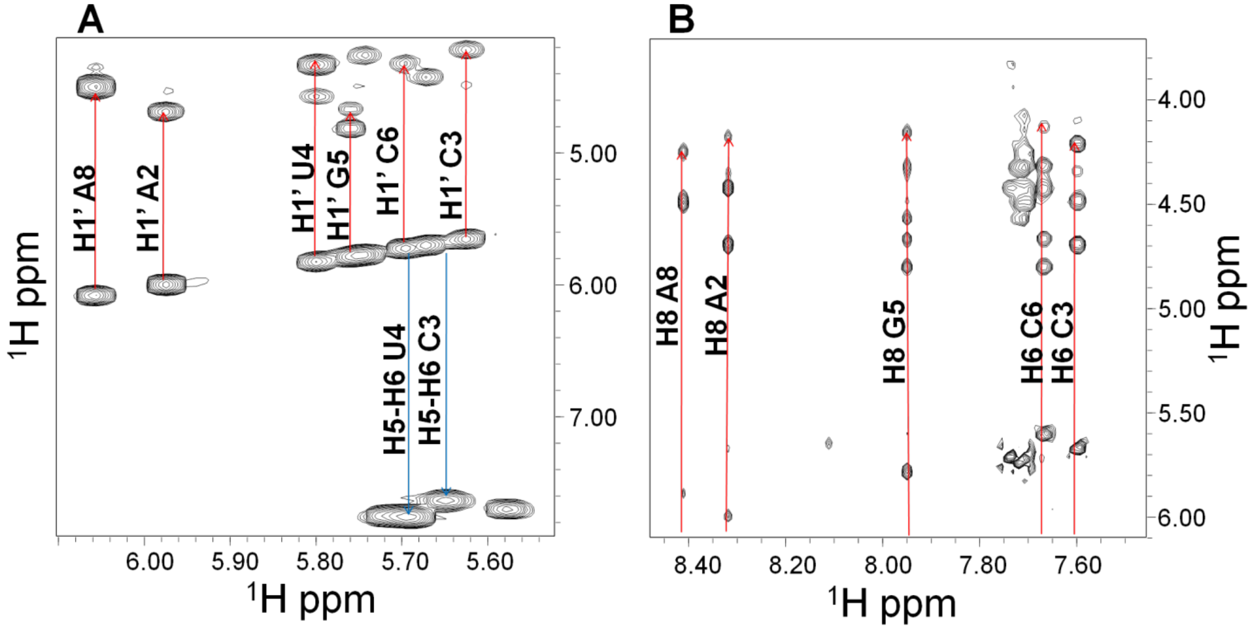

Table S3. ^{1}H chemical shifts (ppm) of PNA1/RNA-1 duplex (unambiguous assignments).

		H6/H8	H2/H5	H1'	H2'	H3'	H4'	H5'/H5''	AMINO	IMINO
RNA	**C1**	8.14	6.10	5.61	4.42	4.63	4.34	3.94, 4.07	7.16, 8.25	
	**A2**	8.22	7.70	6.03	4.72	4.52	4.45			
	**C3**	7.30	5.20	5.51	4.21	4.43	4.31	4.08	6.92, 8.36	
	**U4**	7.73	5.32	5.70	4.57	4.62	4.44	4.12		13.51
	**G5**	7.84		5.89	4.50					12.20
	**C6**	7.52	5.15	5.52	4.38				6.68, 8.61	
	**C7**	7.56	5.42						6.73, 8.57	
	**A8**	8.39		6.06	4.48					

		H6/H8	H2/H5	CH3	2'-3'	8'	HN	AMINO	IMINO
PNA	**PT1**	7.22		1.77	3.32, 3.48, 3.77, 3.85	4.44, 4.55			
	**PG2**	7.62			3.41, 3.78, 3.87				13.18
	**PG3**	6.92			3.39, 3.81, 4.10	4.66, 4.94	8.59		13.22
	**PC4**	6.65	5.35		3.82	3.60, 4.72	8.62	6.81, 8.27	
	**PA5**	7.76	7.06		3.20, 3.47, 3.80, 3.91	4.61, 5.08	8.59		
	**PG6**	6.54			3.20, 3.42, 3.83, 3.91	4.12, 4.62	8.57		13.34
	**PT7**	6.52		1.43	3.14, 3.45, 3.79, 3.88	3.58, 4.79	8.59		
	**PG8**	7.43			3.21, 3.34, 3.77, 3.87	4.39, 4.92	8.40		

Table S4. ^{1}H chemical shifts (ppm) of PNA1/RNA-2 duplex (unambiguous assignments).

		H6/H8	H2/H5	H1'	H2'	H3'	H4'	H5'/H5"	AMINO	IMINO
**RNA**	**C1**	8.17	6.10	5.60	4.43	4.63	4.34	4.05,3.94	7.19, 8.25	
	**A2**	8.24	7.73	6.05	4.71	4.54	4.45			
	**C3**	7.32	5.21	5.51	4.20	4.44	4.32	4.09	6.93, 8.38	
	**U4**	7.76	5.34	5.70	4.57	4.62	4.46	4.11		13.54
	**G5**	7.86		5.90	4.48					12.20
	**C6**	7.53	5.19	5.56	4.37				6.77, 8.67	
	**C7**	7.58	5.46						6.78, 8.63	
	**U8**	7.72	5.57							

		H6/H8	H2/H5	CH3	2'-3'	8'	HN	AMINO	IMINO
**PNA**	**PT1**	7.26		1.77	3.30, 3.47, 3.76, 3.90	4.64, 4.83			
	**PG2**	7.64			3.51, 3.93, 4.02				13.25
	**PG3**	7.03			3.48, 3.81, 4.01	4.62, 5.07	8.67		13.22
	**PC4**	6.78	5.43		3.88	3.70, 4.84	8.73	6.87, 8.33	
	**PA5**	7.83	7.10		3.20, 3.47, 3.80, 3.91	4.69, 5.13	8.67		
	**PG6**	6.57			3.25, 3.45, 3.83, 3.91	4.14, 4.63	8.58		13.34
	**PT7**	6.55		1.44	3.20, 3.45, 3.79, 3.88	3.60, 4.79	8.59		
	**PG8**	7.46			3.25, 3.39, 3.77, 3.87	4.41, 4.95	8.41		

Figure S7. Overlay of TOCSY (red) and NOESY300 (green) spectra of PNA1/RNA-1 **(A, B)** and PNA1/RNA-2 **(C, D)** samples. Spectral regions containing correlations from PNA H_N, RNA and PNA aromatic protons are shown in panels **(A, C)** whereas, the imino/amino-aromatic correlation regions are reported in **(B, D)**.

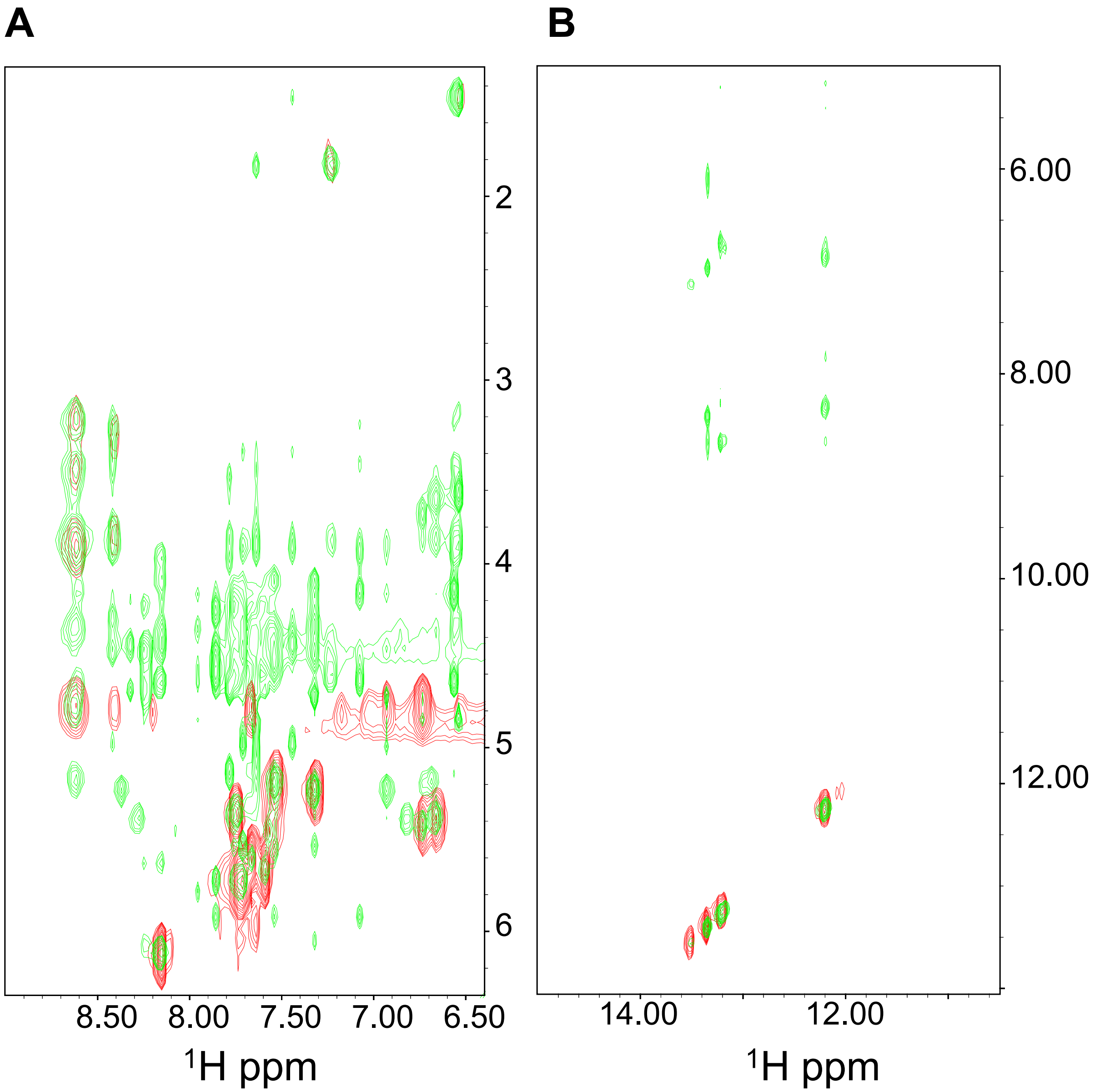

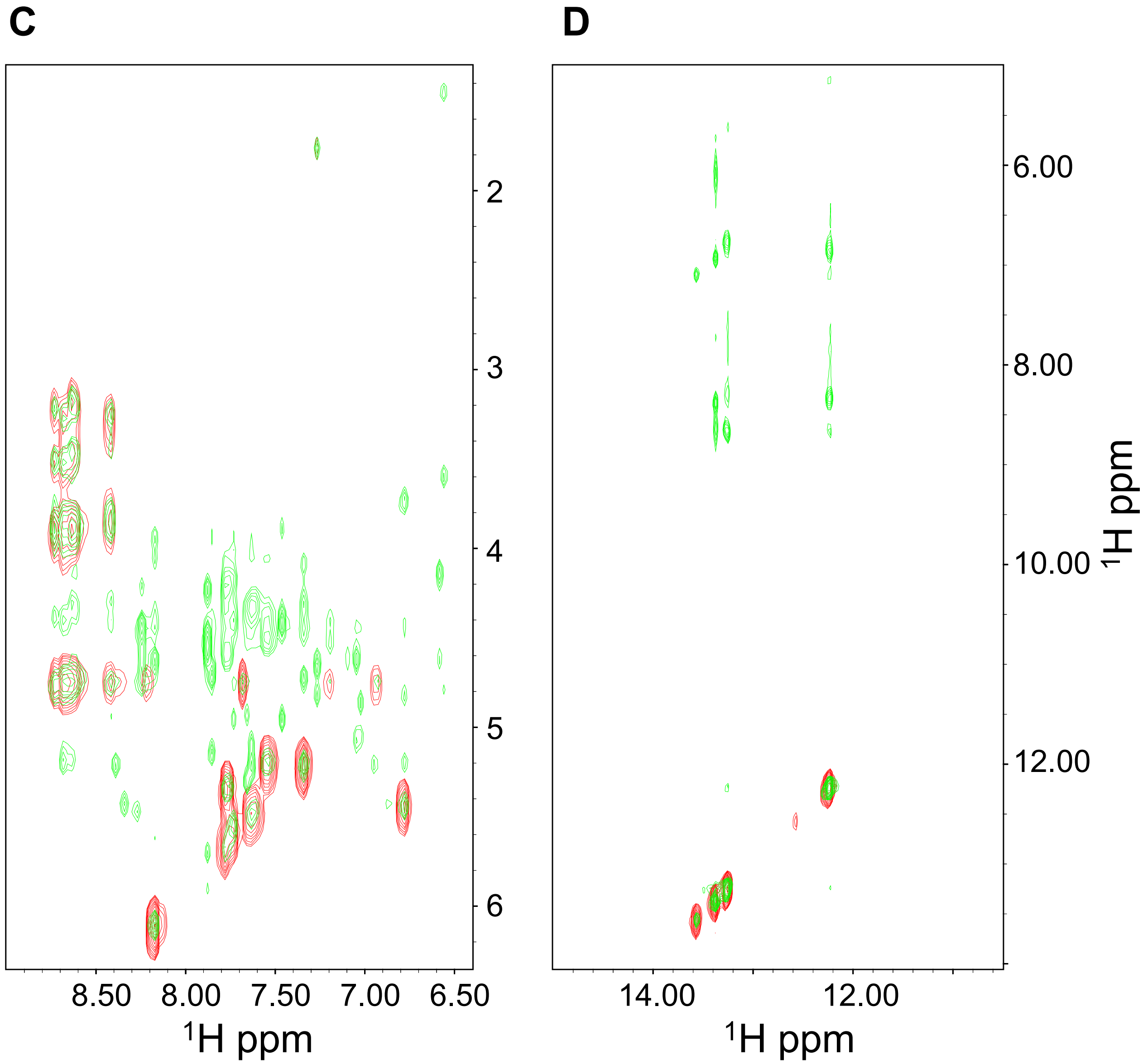
C
D
2
3
4
5
6
8.50
8.00
7.50
7.00
6.50
¹H ppm
14.00
12.00
¹H ppm
6.00
8.00
10.00
12.00
¹H ppm

Figure S8. Superposition of NOESY300 (green) and NOESY200 (red) spectra of the PNA1/RNA-2 sample. Spectral regions containing correlations from PNA H_N, RNA and PNA aromatic protons are shown in the left panel whereas, the imino/amino-aromatic correlation region is shown in the right panel.

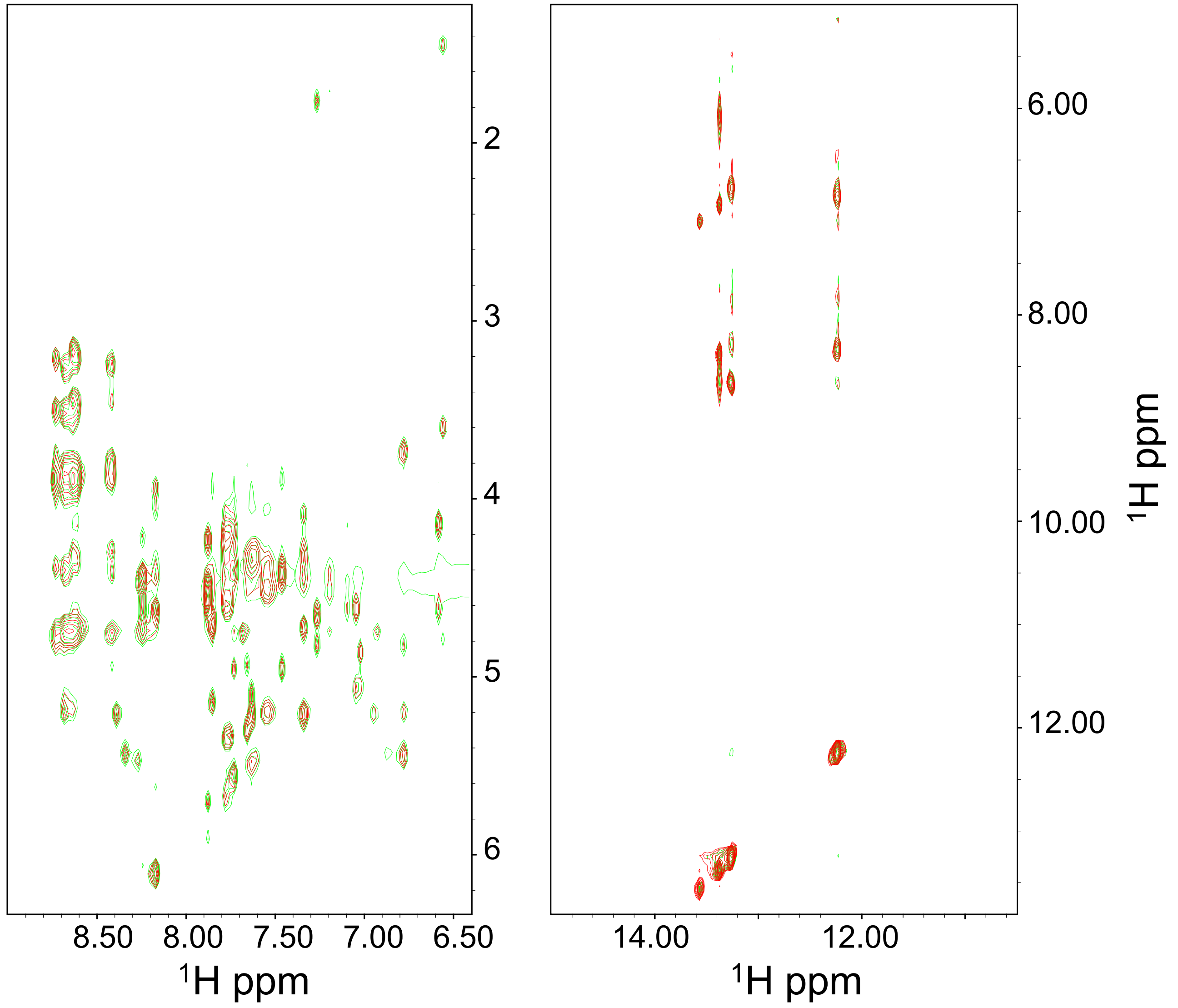

Figure S9. RMSD computed on PNA strand for PNA/RNA-1 duplex, without TPN1 contribution.

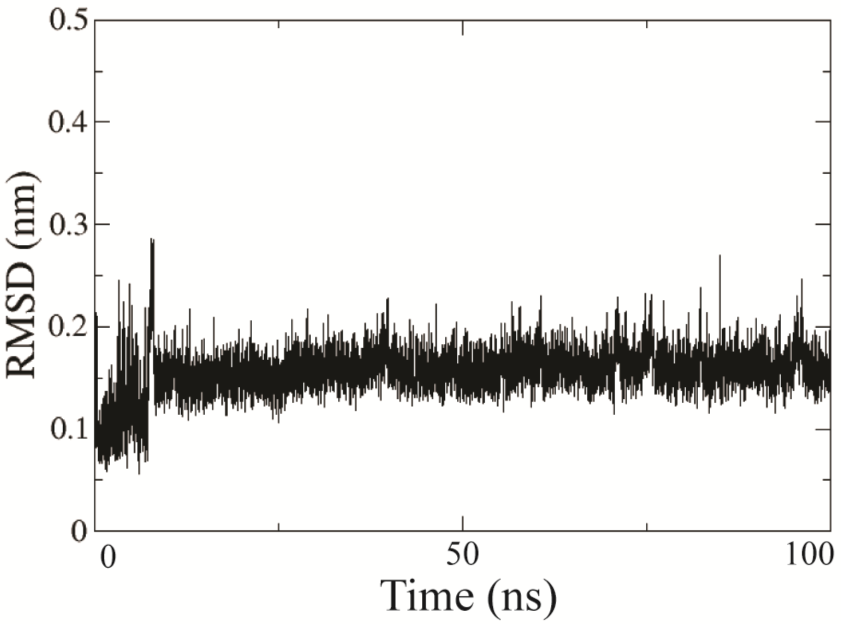

Figure S10. Atom nomenclature used in the computational study through MD simulations.

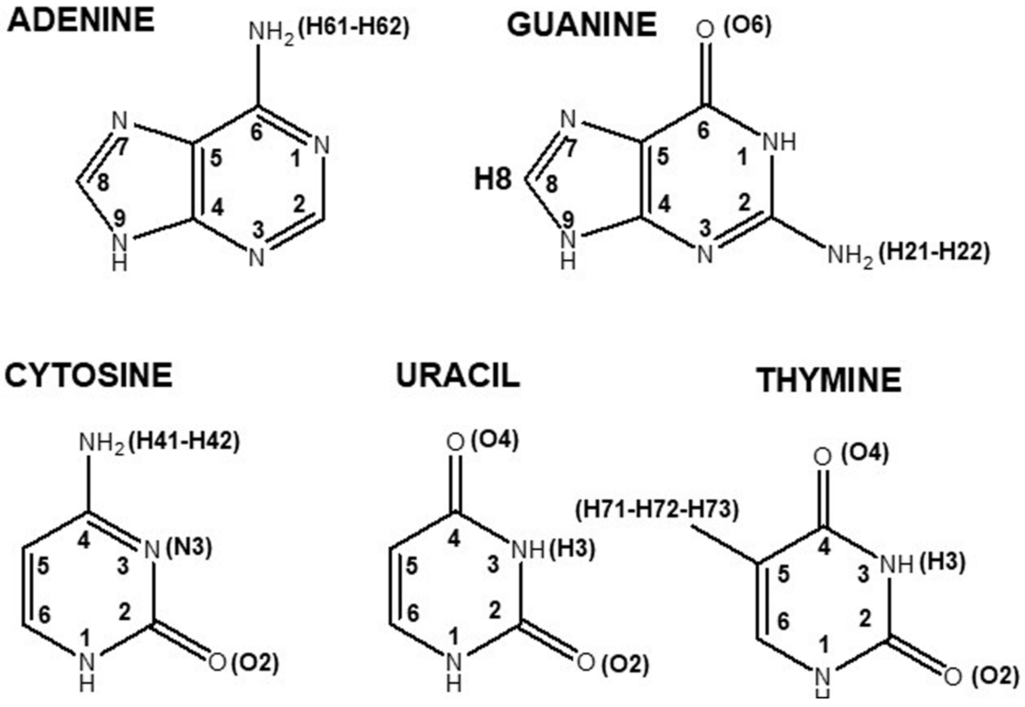

Supplement: Supplementary file 1 [file Data_Sheet_1.PDF]
